# Effects of lifestyle interventions in pregnancy on gestational diabetes: individual participant data and network meta-analysis

**DOI:** 10.1136/bmj-2025-084159

**Published:** 2026-01-07

**Authors:** John Allotey, Dyuti Coomar, Joie Ensor, Gabriel Ruiz-Calvo, Anna Boath, Chidubem Okeke Ogwulu, Mark Monahan, Valencia Kabeya, Min Zheng, Rachel McNeill, Hollie Meacham, Ghadir Mahmoud, Sharon Anne Simpson, Graham A Hitman, Krish Nirantharakumar, Nicola Heslehurst, Mireia Pelaez, Serena Tonstad, SeonAe Yeo, Jose G Cecatti, Fabio Facchinetti, Narges Sadat Motahari-Tabari, Kristina M Renault, Kym J Guelfi, Dorte Møller Jensen, Cheryce Harrison, Mahnaz Bahri Khomami, Alfonso L Calle-Pascual, Fionnuala M McAuliffe, Hans Hauner, Ruben Barakat, Nina Rica Wium Geiker, Christina Anne Vinter, Suzanne Phelan, Tarja I Kinnunen, Alka Kothari, Helena Teede, Lucilla Poston, Ana Pilar Betrán, Ngawai Moss, Stamatina Iliodromiti, Frances Austin, Tracy Roberts, Javier Zamora, Richard D Riley, Shakila Thangaratinam, i-WIP Collaborative Group, Kelly Allison, Ellen Althuizen, Carla Assaf-Balut, Arne Astrup, Erica Baciuk, Isabelle Marc, Annick Bogaerts, José F Cordero, Gustaaf Dekker, Roland Devlieger, Jodie M Dodd, Nermean El Beltagy, María Luisa Garmendia, Kirsti Krohn Garnæs, Isabelle Guelinckx, Lene AH Haakstad, Marquis Hawkins, Amy Hui, Kirby Jeffries, Maria Kennelly, Janette Khoury, Julia Kunath, Riitta Luoto, Elizabeth McCarthy, Siv Mørkved, Fernanda Surita, Simony Lira do Nascimento, Carrie Nobles, Chrsitne M Olson, Ming Jing Ong, Nicolette Oostdam, María Perales, Elisabetta Petrella, Julie Owens, Kathrin Rauh, Kristiina Rono, Jonatan Ruiz, Linda R Sagedal, Kjell Å Salvesen, Tânia T. Scudeller, Alexis Shub, Garry X Shen, Signe N Stafne, Mireille van Poppel, Ingvild Vistad, Jennifer Walsh, Jane Willcox, S Wolff

**Affiliations:** 1 Institute of Life Course and Medical Sciences, University of Liverpool, Liverpool, UK; 2 WHO Collaborating Centre for Global Women’s Health, Department of Metabolism and Systems Science, College of Medicine and Health, University of Birmingham, Birmingham, UK; 3 Department of Applied Health Science, College of Medical and Health, University of Birmingham, Birmingham, UK; 4 National Institute for Health and Care Research (NIHR) Birmingham Biomedical Research Centre, UK; 5 Unidad de Bioestadística Clínica del Hospital Universitario Ramón y Cajal (IRYCIS), Madrid, Spain; 6 Physical Activity for Health Research Centre, University of Edinburgh, Edinburgh, UK; 7 Yunnan Maternal and Child Health Care Hospital, Kunming, Yunnan, China; 8 School of Health and Wellbeing, University of Glasgow, Glasgow, UK; 9 Centre for Genomics and Child Health, Blizard Institute, Faculty of Medicine and Dentistry, Queen Mary University of London, London, UK; 10 School of Life Course and Population Sciences, King’s College London, London, UK; 11 Population Health Sciences Institute, Faculty of Medical Sciences, Newcastle University, Newcastle upon Tyne, UK; 12 Universidad Europea del Atlántico, Santander, Spain; 13 Serena Tonstad, Oslo University Hospital, Oslo, Norway; 14 University of North Carolina at Chapel Hill, School of Nursing, Chapel Hill, NC, USA; 15 Department of Obstetrics and Gynecology, University of Campinas, Campinas, Brazil; 16 University of Modena and Reggio Emilia, Modena, Italy; 17 Faculty of Nursing and Midwifery, Mazandaran University of Medical Sciences, Sari, Iran; 18 Department of Gynecology, Fertility and Obstetrics, Rigshospitalet, University Hospital of Copenhagen, Copenhagen, Denmark; 19 Department of Clinical Medicine, Faculty of Health and Medical Sciences, University of Copenhagen, Copenhagen, Denmark; 20 School of Human Sciences, The University of Western Australia, Perth, Western Australia, Australia; 21 Steno Diabetes Center, Odense University Hospital, Odense, Denmark; 22 Department of Gynaecology and Obstetrics, Odense University Hospital, Odense, Denmark; 23 Department of Clinical Research, Faculty of Health Sciences, University of Southern Denmark, Odense, Denmark; 24 Monash Centre for Health Research and Implementation, School of Public Health, Monash University, Melbourne, Victoria, Australia; 25 Endocrinology and Nutrition Department, Hospital Clínico San Carlos and Instituto de Investigación Sanitaria del Hospital Clínico San Carlos (IdISSC), Madrid, Spain; 26 Centro de Investigación Biomédica en Red de Diabetes y Enfermedades Metabólicas Asociadas (CI-BERDEM), Madrid, Spain; 27 UCD Perinatal Research Centre, University College Dublin, National Maternity Hospital, Dublin, Ireland; 28 Institute of Nutritional Medicine, School of Medicine and Health, Technical University of Munich, Munich, Germany; 29 Universidad Politécnica de Madrid, Madrid, Spain; 30 The Centre for Childhood Health, Nono Nordisk, Denmark; 31 Center for Health Research, California Polytechnic State University, San Luis Obispo, CA, USA; 32 Unit of Health Sciences, Faculty of Social Sciences, Tampere University, Tampere, Finland; 33 Redcliffe Hospital, Redcliffe, Queensland, Australia; 34 Faculty of Medicine, The University of Queensland, St Lucia, Queensland, Australia; 35 Division of Women’s Health, Women’s Health Academic Centre, King’s College London, St Thomas’ Hospital, London, UK; 38 Department of Reproductive and Health Research, World Health Organization, Geneva, Switzerland; 37 Katies Team, Patient and Public Representative, London, UK; 38 Wolfson Institute of Population Health, Faculty of Medicine and Dentistry, Queen Mary University of London, London, UK; 39 Women's and Children's Health Services, Barts Health NHS Trust, London, UK; 40 CIBER Epidemiology and Public Health (CIBERESP), Madrid, Spain; 41 Liverpool Women's NHS Foundation Trust, Liverpool, UK; 42 NIHR Northwest Coast Applied Research Collaboration, Liverpool, UK

## Abstract

**Objectives:**

To assess the effects of lifestyle interventions on gestational diabetes, determine whether the effects vary by maternal body mass index, age, parity, ethnicity, education level, or intervention, and rank interventions by effectiveness.

**Design:**

Individual participant data (IPD) and network meta-analysis.

**Data sources:**

Major electronic databases (January 1990 to April 2025).

**Methods:**

This meta-analysis included randomised trials on the effects of lifestyle interventions (physical activity based, diet based, or mixed) in pregnancy on gestational diabetes. Main outcomes were gestational diabetes defined by any criteria and by UK NICE (National Institute for Health and Care Excellence) criteria; other outcomes included IADPSG (International Association of Diabetes in Pregnancy Study Group) and modified IADPSG defined gestational diabetes. A two stage IPD meta-analysis estimated summary odds ratios and 95% confidence intervals and interactions (subgroup effects), along with absolute risk reduction estimates. Aggregate data from non-IPD trials were added to the meta-analysis when possible. Intervention effects were ranked using network meta-analysis.

**Results:**

104 randomised trials (35 993 women) were included, with IPD for 68% of participants (24 391 women; 54 studies). Lifestyle interventions reduced gestational diabetes defined by any criteria by 10% in IPD trials (odds ratio 0.90, 95% confidence interval (CI) 0.80 to 1.02; absolute risk reduction 1.3%, 95% CI −0.3% to 2.6%), and by 20% when combining IPD and non-IPD trials (odds ratio 0.80, 95% CI 0.73 to 0.88; absolute risk reduction 2.6%, 95% CI 1.6% to 3.6%), and no reduction was observed using NICE criteria (odds ratio 0.98, 95% CI 0.84to 1.13). Lifestyle interventions reduced gestational diabetes defined using IADPSG criteria by 14% in IPD trials (odds ratio 0.86, 95% CI 0.75 to 0.97; absolute risk reduction 2.7%, 95% CI 0.6% to 5.0%) and by 18% when combining IPD and non-IPD trials (odds ratio 0.82, 95% CI 0.72 to 0.93; absolute risk reduction 3.5%, 95% CI 1.3% to 5.7%). Effects did not vary by maternal characteristics, except for education. Although women of all educational levels benefited from the intervention, the benefit was less in those with low education (low *v* middle interaction: odds ratio 0.68, 95% CI 0.51 to 0.90; low *v* high interaction: odds ratio 0.71, 95% CI 0.54 to 0.93). Benefits did not vary by intervention characteristics, except for greater effectiveness with group format (odds ratio 0.81, 95% CI 0.68 to 0.97; absolute risk reduction 2.5%, 95% CI 0.4% to 4.3%) and newly trained facilitators (odds ratio 0.82, 95% CI 0.69 to 0.96; absolute risk reduction 2.4%, 95% CI 0.5% to 4.2%). Physical activity based interventions ranked highest (mean rank 1.1, 95% CI 1 to 2) in preventing gestational diabetes.

**Conclusions:**

Lifestyle interventions in pregnancy are likely to prevent gestational diabetes, with effects varying according to diagnostic criteria. Implementation strategies should address inequalities by maternal education, and consider group formats, provider training, and physical activity based interventions to prevent gestational diabetes.

**Study registration:**

PROSPERO CRD42020212884.

## Introduction

Gestational diabetes, characterised by glucose intolerance first diagnosed during pregnancy, affects 7-38% of pregnancies worldwide.^1^ Gestational diabetes poses substantial risks to mother and baby during pregnancy because of increased risk of stillbirths, preterm births, pre-eclampsia, caesarean section, large for gestational fetuses, and birth trauma.^2^ ^3^ In the long term, gestational diabetes predisposes the mother and her offspring to obesity, type 2 diabetes, and cardiovascular complications.^2^ ^4^ The rates of gestational diabetes are rising worldwide owing to a population level increase in sedentary behaviour, poor diet, and obesity; these rates need to be curbed.^5^ Lifestyle interventions such as physical activity and dietary modifications that are effective in preventing type 2 diabetes^6^ have the potential to prevent gestational diabetes.

Despite the investment of over £10m ($13.1m; €11.3m) in trials on lifestyle interventions in pregnancy, none have been implemented in routine practice.^7^ ^8^ ^9^ Randomised trials and systematic reviews report clear benefits of lifestyle interventions in pregnancy in reducing gestational weight gain,^7^ ^8^ but findings vary for gestational diabetes.^7^ ^9^ ^10^ ^11^ Robust evidence is lacking to guide policy makers in making recommendations on the preferred type of lifestyle intervention to prevent gestational diabetes, or whether the interventions should be focused on specific groups of pregnant women. Study level meta-analyses using aggregate data are limited by the heterogeneity in the reported study populations, interventions, and outcome definitions.^10^ We also do not know if the effects of interventions on gestational diabetes vary by maternal characteristics, such as body mass index, age, parity, ethnicity, and socioeconomic status, or by components of the intervention.^12^ ^13^

In this individual participant data (IPD) meta-analysis of randomised trials, we firstly assessed the effects of lifestyle interventions categorised as mainly physical activity based, diet based, or with mixed components, on gestational diabetes defined by any criteria and by UK NICE (National Institute for Health and Care Excellence) criteria. Secondly, we assessed these effects using the IADPSG (International Association of Diabetes in Pregnancy Study Group) and modified IADPSG criteria, reflecting international variation in diagnostic thresholds, clinical guidelines, and healthcare practices.^14^ We studied whether the intervention effects varied by baseline maternal body mass index, age, parity, ethnicity, or education level, and by intervention components. We ranked the interventions by their effectiveness in reducing gestational diabetes and assessed their effects on critically important maternal and perinatal outcomes.

## Methods

We undertook the IPD meta-analysis using a prospective protocol registered with PROSPERO (CRD42020212884),^12^ and reported in line with recommendations of the PRISMA-IPD (preferred reporting items for systematic reviews and meta-analysis of individual participant data) guidelines.^15^

### Study governance and data source

The IPD were provided by members of the i-WIP Collaborative Group.^8^ Relevant trials were identified by a systematic review of the literature. We have previously reported details on how we contacted the authors and obtained data that were checked for quality, recoded, and harmonised for analyses.^8^ Briefly, eligible trials were identified through systematic searches of major electronic databases, supplemented by internet searches and contact with research experts. We established the i-WIP Collaborative Group by contacting researchers of eligible studies and asking them to share data in any format along with data dictionaries or coding guides. A bespoke database was developed for the IPD, and data were checked for completeness, plausibility, and consistency against published reports. Data were then formatted, recoded, and harmonised across trials to enable participant level analyses. Full details of these procedures are available in our previous publications.^8^ ^13^ The i-WIP data sharing committee approved the use of the data. An independent project steering committee oversaw the conduct of the study. University of Birmingham Research Ethics (ERN_20-1748) confirmed exemption from formal ethics approval.

### Search strategy and study selection

We updated our previous systematic review using two search periods to identify new trials on diet and physical activity in pregnancy.^13^ In the first period (from February 2017 to March 2021, which was the endpoint for IPD acquisition to allow sufficient time for data cleaning, standardisation, and amalgamation of datasets), we identified trials to obtain IPD to add to our existing i-WIP IPD repository. We undertook a further search in the second period (from April 2021 to April 2025) to identify new trials published after the IPD acquisition timeline. We searched Medline, Embase, BIOSIS, LILACS, Pascal, Science Citation Index, Cochrane Database of Systematic Reviews, Cochrane Central Register of Controlled Trials, Database of Abstracts of Reviews of Effects and Health Technology Assessment Database without language restrictions. Supplementary web appendix 1 provides details of the search strategy. Two independent reviewers (DC and AB) performed the study selection process, with disagreements resolved by a third reviewer (JA).

We included trials that randomly assigned pregnant women as individuals or in clusters to lifestyle interventions (physical activity, diet, mixed) or standard care and collected relevant data on gestational diabetes. We excluded women with a known diagnosis of gestational diabetes at baseline or trials that evaluated weight loss interventions such as surgery or pharmacotherapy. Lifestyle interventions were grouped into mainly physical activity based interventions that were supervised or non-supervised; mainly diet based interventions involving a specific diet like the Mediterranean diet or other supervised and non-supervised dietary plans; and mixed interventions providing overall guidance on diet and physical activity with varying levels of intensity and structure.^12^

The primary outcomes were gestational diabetes as defined by any criteria and by NICE criteria.^2^ Secondary outcomes included gestational diabetes defined by IADPSG criteria,^16^ and critically important maternal and perinatal outcomes previously determined by a Delphi survey.^17^ Supplementary web appendix 2 provides the outcome definitions. We invited the authors of relevant studies identified in the first search period to join the i-WIP Collaborative Group and share participant level data with the i-WIP database in any format. When there was no initial response, we sent three further reminders to each author. For studies that did not provide IPD or whose authors did not respond, or those included in the second search period, we extracted the published aggregate data.

### Quality assessment and data extraction

Two independent reviewers (DC and AB) assessed the quality of the included studies using the Cochrane risk of bias tool for sequence generation, allocation concealment, blinding, completeness of outcome data, and selective outcome reporting.^18^ We evaluated outcome selective reporting by confirming whether gestational diabetes was a prespecified outcome and whether it was fully reported. We considered a study to have a high risk of bias if any of the following domains were considered to be at high risk: randomisation, allocation concealment, blinding of outcome assessment, and completeness of outcome data. These domains should be scored as low risk for a study to be classified as low risk of bias. For trials that shared IPD, we used the IPD to assess for selection bias by evaluating between-group baseline imbalances for the key prognostic factors like age and body mass index, and for attrition bias by studying the completeness of outcome data for each woman in each group. Two independent researchers (DC and AB, VK, GM, or MBK) undertook data extraction at the study level for inclusion and exclusion criteria, the characteristics of the intervention, and the reported outcomes. We used the Template for Intervention Description and Replication (TIDieR) framework^19^ to map and categorise the core components of lifestyle interventions. We also extracted the published study level data for studies published beyond the IPD acquisition phase, and those for which IPD were not provided by the study authors.

### Statistical analysis

We performed a two stage IPD meta-analysis to obtain summary estimates of the odds ratio and 95% confidence interval (CI) for the overall intervention effect, and for the interaction between potential effect modifiers (baseline body mass index, age, parity, ethnicity, or education level) and intervention effect for each primary outcome. We assessed the overall effects of lifestyle interventions and by each intervention type (physical activity based, diet based, and mixed approach). Participant level missing data patterns and baseline imbalances were summarised to check for systematic differences in missing data, as detailed in our statistical analysis plan (supplementary web appendix 3). All analyses were performed after imputing a minimal subset of missing data using the corresponding mean of participants within the same study and allocation group.

For the two stage analysis of the overall intervention effect, in the first stage, we fitted logistic regression models for each trial separately with the intervention as a covariate, adjusting for maternal age and body mass index where available. For cluster trials, we additionally included a random effect for the unit of randomisation (to account for clustering). For trials with several intervention arms, we analysed each intervention separately when these were different, or combined groups when they were similar, with all comparisons made against usual care. In the second stage, we pooled the studies intervention effect estimates using a random effects meta-analysis model fitted with restricted maximum likelihood. Confidence intervals for the summary effect were inflated using the Hartung-Knapp correction.^20^ To aid interpretation, we calculated absolute risk reductions and their 95% CIs by applying the pooled odds ratios to the average baseline risk of the outcome across all studies included in the meta-analysis, following GRADE (grading of recommendations assessment, development and evaluation) guidance.^21^ We investigated small study effects (potential publication or availability bias)^22^ through contour enhanced funnel plots for analyses containing 10 or more studies. We obtained summary estimates of overall intervention effects across all published studies by incorporating the study level data of studies that did not share IPD within the second stage of the IPD meta-analysis framework. Sensitivity analyses were conducted to exclude IPD from studies at high risk of bias. Heterogeneity was summarised using the estimated between study variance (τ^2^) and by approximate 95% prediction intervals for the intervention (or interaction) effect in a new study.^23^

To assess the differential effects of the intervention by maternal characteristics, we extended the models to include treatment covariate interaction terms for maternal body mass index, age, parity, ethnicity, and education level in the IPD studies only. We obtained summary estimates of these subgroup effects (interactions) using the two stage IPD meta-analysis framework for the overall intervention. Interaction effects were first estimated within each study by fitting a regression model that included the intervention group, the potential effect modifier (subgroup variable), and their interaction term. The coefficient of the interaction term (on the log odds scale) was extracted from each study and then pooled in a random effects meta-analysis model in the second stage to obtain a summary interaction effect. Continuous covariates were analysed on their continuous scale and for predetermined, clinically defined, categorical values. To assess the differential effects by TIDieR intervention components, we conducted random effects meta-regression analyses using study level effect estimates. Intervention characteristics, including theory basis, resource provision, facilitator type, facilitator training, mode and structure of delivery, setting, number and duration of sessions, and gestational age at intervention start, were included as explanatory variables in separate meta-regression models.

For secondary binary outcomes, we used logistic regression models in the first stage and random effects meta-analysis in the second stage to obtain summary estimates and 95% CIs for the intervention effects (odds ratios). Forest plots were generated to display study specific and pooled results. To combine direct and indirect evidence to estimate intervention effects, we performed a network meta-analysis for gestational diabetes defined by any criteria using a multivariate random effects framework.^24^ We were unable to statistically test the consistency assumption owing to the geometry of the network. Finally, we calculated the intervention rankings using resampling methods and displayed these graphically.^25^ We used Stata MP version 18.0 for analysis and analysis code is available in the https://github.com/JoieEnsor/iWIP-GDM-project repository.

### Patient and public involvement

Members of the public were involved in prioritising the research question, and developing, designing, and managing the research. The study was supported by The Hildas (https://www.dhlnetwork.com/news), a dedicated patient and public involvement group in women’s health. The team members were involved in the interpretation and reporting of the results.

## Results

**Fig 1:**
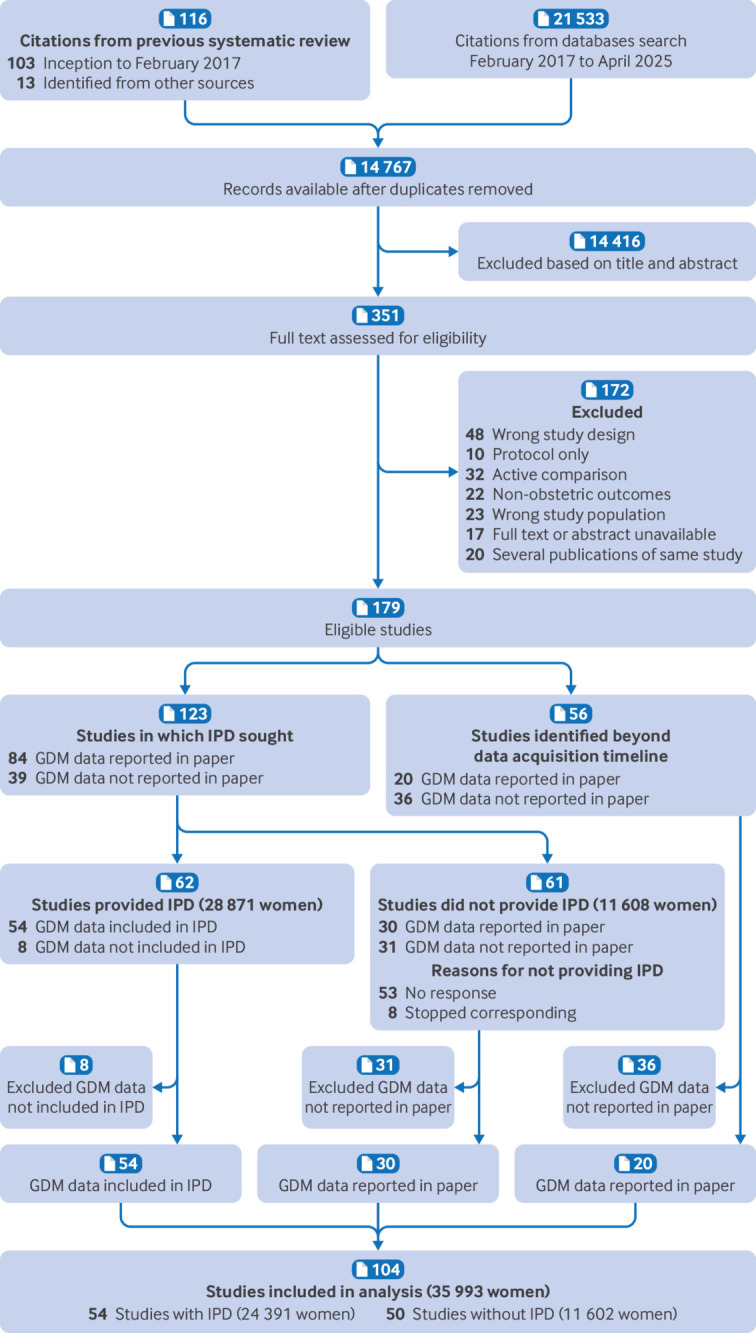
Identification and selection of studies included in individual participant data (IPD) meta-analysis of effects of lifestyle interventions on gestational diabetes (GDM)

We included 104 randomised trials involving 35 993 women. Individual participant data were available for 68% of all participants (24 391 women, 54 trials). Fifty trials (11 602 women) did not have IPD available and contributed only aggregate data (fig 1).

### Characteristics of included studies

Overall, 48 trials were conducted in Europe (33/48 shared IPD), 24 in North America (9/24 shared IPD), 10 in Australia (8/10 shared IPD), and 6 in South America (4/6 shared IPD). Of the 54 trials that shared IPD, most were randomised trials with individual participant allocation (51/54, 94%), while three were cluster randomised trials.^26^ ^27^ ^28^ In the studies that contributed IPD, participants had an average age of 29 years (standard deviation 6.0), 81% were white, 50% were nulliparous, 49% held a higher education degree, and 10% had a previous diagnosis of gestational diabetes (table 1). Eleven IPD trials included only women with obesity,^9^ ^29^ ^30^ ^31^ ^32^ ^33^ ^34^ ^35^ ^36^ ^37^ ^38^ 10 included both women with obesity and those who were overweight,^39^ ^40^ ^41^ ^42^ ^43^ ^44^ ^45^ ^46^ ^47^ ^48^ four studies included only overweight women,^27^ ^49^ ^50^ ^51^ and 29 included women of any body mass index.^11^ ^26^ ^28^ ^52^ ^53^ ^54^ ^55^ ^56^ ^57^ ^58^ ^59^ ^60^ ^61^ ^62^ ^63^ ^64^ ^65^ ^66^ ^67^ ^68^ ^69^ ^70^ ^71^ ^72^ ^73^ ^74^ ^75^ ^76^ ^77^ The physical activity based interventions included water aerobics, fitness sessions or exercise programmes, and strength training with or without trainer supervision in 18 IPD trials (36 total).^29^ ^31^ ^42^ ^47^ ^48^ ^54^ ^55^ ^56^ ^57^ ^59^ ^60^ ^65^ ^69^ ^70^ ^71^ ^73^ ^75^ ^78^ Diet based interventions included the Mediterranean diet, a cholesterol lowering diet, and basic dietary advice on gestational weight gain in eight IPD trials (18 total),^35^ ^38^ ^46^ ^53^ ^61^ ^66^ ^76^ ^77^ and a mixed approach involving advice on physical activity, diet, or behaviour changing techniques in 28 IPD trials (52 total).^9^ ^11^ ^26^ ^27^ ^28^ ^30^ ^32^ ^33^ ^34^ ^36^ ^37^ ^39^ ^40^ ^41^ ^43^ ^44^ ^45^ ^49^ ^50^ ^51^ ^52^ ^62^ ^63^ ^64^ ^67^ ^68^ ^72^ ^74^ Three trials had a three arm design, with intervention arms being different types of counselling or diet, or different exercise routines.^30^ ^33^ ^35^ Fifty four trials (23 361 women) provided IPD on gestational diabetes as defined by any criteria (total 104 studies, 35 541 women), 22 IPD trials (11 990 women) according to NICE criteria (total 23 studies, 12 041 women), and 16 IPD trials (6174 women) according to IADPSG criteria (total 29 studies, 8626 women). Supplementary web appendix 4 provides the characteristics of all IPD studies included in the meta-analysis and studies contributing aggregate data only. Supplementary web appendix 5 provides components of the interventions of all IPD studies classified using the TIDieR framework.^19^

**Table 1:** Baseline characteristics of women included in individual participant data meta-analysis of effects of lifestyle interventions on gestational diabetes

Baseline characteristics	No of studies (No of women)	Study arm	
		Control (n=11 160)	Intervention (n=12 538)
Age (years), mean (SD)	53 (23 607)	29.5 (6.0)	29.4 (6.0)
<20	—	548 (4.9)	649 (5.2)
≥20	—	10 566 (95.1)	11 844 (94.8)
Height (cm), mean (SD)	51 (21 560)	163.3 (7.1)	163.5 (7.0)
Weight (kg), mean (SD)	38 (15 977)	78.6 (18.4)	78.2 (18.2)
Body mass index, mean (SD)	54 (23 698)	28.0 (6.2)	27.9 (6.2)
Normal	—	4330 (38.8)	4898 (39.1)
Overweight	—	2977 (26.7)	3385 (27.0)
Obese	—	3853 (34.5)	4255 (33.9)
Race or ethnicity	35 (12 649)	—	—
White	—	4995 (80.7)	5294 (81.9)
Asian	—	497 (8.0)	488 (7.6)
Black	—	407 (6.6)	398 (6.2)
Central or South American	—	87 (1.4)	77 (1.2)
Middle Eastern	—	79 (1.3)	75 (1.2)
Other	—	122 (2.0)	130 (2.0)
Educational status of mother†	35 (11 719)	—	—
Low	—	1108 (19.5)	997 (16.5)
Middle	—	1881 (33.2)	1979 (32.7)
High	—	2682 (47.3)	3072 (50.8)
Smoking status	—	—	—
Current smoker	44 (18 330)	842 (9.6)	851 (8.9)
Previous smoker (before pregnancy)	24 (9969)	1494 (32.3)	1624 (30.4)
Gestational age at randomisation, mean (SD)	39 (18 820)	12.5 (4.6)	12.5 (4.2)
Parity	45 (21 561)	—	—
0	—	4931 (48.7)	5836 (50.6)
1	—	3317 (33.1)	3704 (32.1)
2	—	1180 (11.8)	1328 (11.5)
3	—	376 (3.7)	429 (3.7)
>4	—	231 (2.3)	229 (2.0)
Underlying medical condition	—	—	—
Previous gestational diabetes	19 (5802)	289 (9.9)	287 (10.0)
Previous hypertension in pregnancy	41 (17 926)	914 (10.8)	1015 (10.8)
Chronic hypertension	41 (5654)	50 (2.1)	76 (2.4)

Values are numbers (percentages) unless stated otherwise. SD=standard deviation.

Low=not completed secondary education to A level; medium=completed secondary education (A level equivalent); high=any further or higher education.

### Quality of included studies

**Fig 2:**
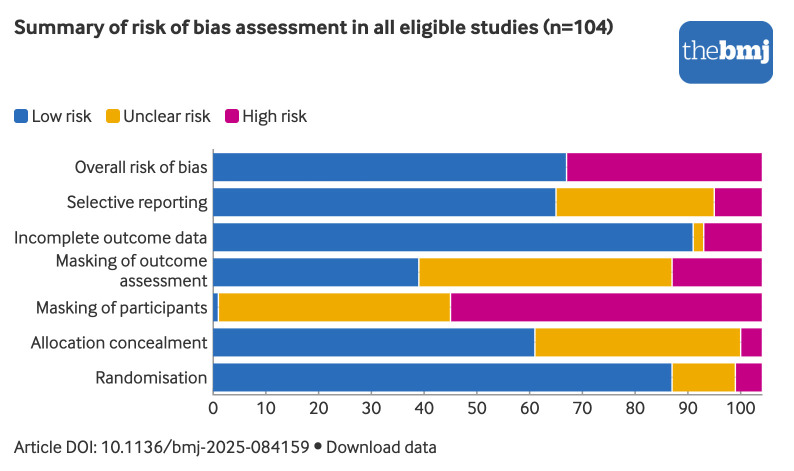
Summary of risk of bias assessment in all eligible studies (n=104). An interactive version of this graphic and downloadable data are available at https://public.flourish.studio/visualisation/26236883/

The global risk of bias was low in about two thirds of all eligible studies (64%, 67/104) (supplementary web appendix 6). More IPD studies had low risk of bias for random sequence generation than those without IPD availability (91% *v* 76%), allocation concealment (61% *v* 56%), masking of outcome assessment (41% *v* 34%), and completeness of outcome data (89% *v* 86%). Figure 2 shows the summary of the risk of bias rating by domain for all eligible studies.

### Effects on gestational diabetes

#### Gestational diabetes defined by any criteria

Our IPD meta-analysis of overall lifestyle interventions showed a 10% reduction in the odds of gestational diabetes (odds ratio 0.90, 95% CI 0.80 to 1.02, τ^2^=0.04, 54 studies, 23 361 women) with an absolute risk reduction of 1.3% (95% CI −0.3% to 2.6%) equivalent to 13 fewer women with gestational diabetes per 1000 women (95% CI 26 fewer to 3 more). Addition of aggregate data from the non-IPD trials (12 180 women, 50 trials) to the meta-analysis resulted in a larger reduction in the odds of gestational diabetes (odds ratio 0.80, 95% CI 0.73 to 0.88, τ^2^=0.07, 104 studies, 35 541 women; table 2), absolute risk reduction 2.6% (95% CI 1.6% to 3.6%) equivalent to 26 fewer women with gestational diabetes per 1000 women (95% CI 36 fewer to 16 fewer). The beneficial effect of overall lifestyle interventions remained when we excluded IPD and non-IPD trials at high risk of bias in the sensitivity analysis (odds ratio 0.87, 95% CI 0.78 to 0.97, τ^2^=0.05, 68 studies, 24 566 women), but not when high risk of bias IPD trials alone were excluded (supplementary web appendix 7).

**Table 2:** Effects of lifestyle interventions on gestational diabetes defined by any criteria and NICE criteria summarised using IPD alone and by supplementing IPD with study level data from studies that did not contribute IPD

Intervention and source	No of studies (No of women)	Odds ratio (95% CI)	95% PI	τ^2 ^(95% CI)
**Any criteria**				
Physical activity				
IPD only	18 (4435)	0.64 (0.48 to 0.84)	0.39 to 1.05	0.04 (0.00 to 0.43)
IPD and aggregate data	36 (9683)	0.64 (0.53 to 0.76)	0.42 to 0.97*	0.30 (0.00 to 0.23)
Diet				
IPD only	8 (2974)	0.81 (0.69 to 0.96)	0.68 to 0.97*	0.00 (0.00 to 0.17)
IPD and aggregate data	18 (5144)	0.78 (0.62 to 0.99)	0.44 to 1.38	0.06 (0.00 to 0.35)
Mixed				
IPD only	28 (15 952)	1.05 (0.91 to 1.21)	0.78 to 1.40	0.02 (0.00 to 0.12)
IPD and aggregate data	52 (20 714)	0.92 (0.82 to 1.04)	0.60 to 1.41	0.04 (0.00 to 0.14)
All				
IPD only	54 (23 361)	0.90 (0.80 to 1.02)	0.59 to 1.38	0.04 (0.01 to 0.13)
IPD and aggregate data	104 (35 541)	0.80 (0.73 to 0.88)	0.47 to 1.37	0.07 (0.03 to 0.15)
**NICE criteria**				
Physical activity				
IPD only	5 (977)	0.65 (0.18 to 2.31)	0.04 to 11.49	0.60 (0.00 to 8.8)
IPD and aggregate data	5 (977)	0.65 (0.18 to 2.31)	0.04 to 11.49	0.60 (0.00 to 8.8)
Diet				
IPD only	3 (1812)	0.70 (0.33 to 1.49)	0.08 to 6.47	0.00 (0.00 to 2.8)
IPD and aggregate data	4 (1863)	0.72 (0.41 to 1.27)	0.33 to 1.55	0.00 (0.00,2.68)
Mixed				
IPD only	14 (9201)	1.10 (0.98 to 1.23)	0.99,1.24	0.00 (0.00 to 0.06)
IPD and aggregate data	14 (9201)	1.10 (0.98 to 1.23)	0.99,1.24	0.00 (0.00 to 0.06)
All				
IPD only	22 (11 990)	0.98 (0.84 to 1.13)	0.70 to 1.36	0.02 (0.00 to 0.13)
IPD and aggregate data	23 (12 041)	0.98 (0.85 to 1.14)	0.71 to 1.33	0.017(0.00 to 0.12)

CI=confidence interval; IPD=individual participant data; NICE=National Institute for Health and Care Excellence; PI=prediction interval.

Among the types of interventions, IPD meta-analysis showed reductions in gestational diabetes with physical activity based (odds ratio 0.64, 95% CI 0.48 to 0.84, τ^2^=0.04, 18 studies, 4435 women; absolute risk reduction 4.9%, 95% CI 2.1% to 7.2%) and diet based interventions (odds ratio 0.81, 95% CI 0.69 to 0.96, τ^2^=0.00, eight studies, 2974 women; absolute risk reduction 2.5%, 95% CI 0.51% to 4.2%), but not with mixed interventions (odds ratio 1.05, 95% CI 0.91 to 1.21, τ^2^=0.02, 28 studies, 15 952 women). We observed the beneficial effects to persist for physical activity based (odds ratio 0.64, 95% CI 0.53 to 0.76, τ^2^=0.03, 36 studies, 9683 women; absolute risk reduction 4.9%, 95% CI 3.2% to 6.4%) and diet based interventions (odds ratio 0.78, 95% CI 0.62 to 0.99, τ^2^=0.06, 18 studies, 5144 women; absolute risk reduction 2.9%, 95% CI 0.1% to 5.1%) when non-IPD trials were added (table 2). The beneficial effect for physical activity based interventions remained when we removed high risk of bias IPD studies (odds ratio 0.59, 95% CI 0.43 to 0.82, τ^2^=0.00, 11 studies, 2993 women), and high risk of bias non-IPD studies (odds ratio 0.67, 95% CI 0.55 to 0.82, τ^2^=0.00, 22 studies, 6967 women) from the analyses, but the findings varied for diet based and mixed interventions (supplementary web appendix 7).

#### Gestational diabetes defined by NICE criteria

Lifestyle interventions did not reduce the odds of gestational diabetes defined by NICE criteria in the IPD meta-analysis (odds ratio 0.98, 95% CI 0.84 to 1.13, τ^2^=0.02, 22 studies, 11 990 women; absolute risk reduction 0.3%, 95% CI −1.6% to 2.1%) or when non-IPD trials were added to the IPD meta-analysis (odds ratio 0.98, 95% CI 0.85 to 1.14, τ^2^=0.02, 23 studies, 12 041 women; absolute risk reduction 0.3%, 95% CI −1.8% to 2.0%). Because of wide confidence intervals, it remains unclear whether reductions in gestational diabetes occur for specific interventions such as physical activity (odds ratio 0.65, 95% CI 0.18 to 2.31, τ^2^=0.60, five studies, 977 women; absolute risk reduction 4.7%, 95% CI −14.0% to 11.9%) and diet (odds ratio 0.71, 95% CI 0.33 to 1.49, τ^2^=0.00, three studies, 1812 women; absolute risk reduction 3.9%, 95% CI −5.8% to 9.5%), although reductions from using mixed interventions are unlikely (odds ratio 1.10, 95% CI 0.98 to 1.23, τ^2^=0.00, 14 studies, 9201 women; table 2). Findings are similar from the sensitivity analyses that excluded high risk of bias studies (supplementary web appendix 7).

#### Gestational diabetes defined by IADPSG and modified IADPSG criteria

The odds of gestational diabetes defined by IADPSG criteria were reduced by lifestyle interventions compared with usual care in the IPD meta-analysis (odds ratio 0.86, 95% CI 0.75 to 0.97, τ^2^=0.00, 16 studies, 6174 women) with an absolute risk reduction of 2.7% (95% CI 0.6% to 5.0%), equivalent to 27 fewer women with gestational diabetes per 1000 women (95% CI 50 fewer to 6 fewer) for a 25% baseline risk of gestational diabetes when using the IADPSG criteria. The reduction persisted when non-IPD trials were added to the analysis (odds ratio 0.82, 95% CI 0.72 to 0.93, τ^2^=0.01, 29 studies, 8626 women; absolute risk reduction 3.5%, 95% CI 1.3% to 5.7%; table 3). Among individual interventions, a reduction in IADPSG defined gestational diabetes was observed for mixed interventions (odds ratio 0.83, 95% CI 0.71 to 0.96, τ^2^=0.00, 17 studies, 5892 women; absolute risk reduction 3.3%, 95% CI 0.8% to 5.9%) when non-IPD trials were added to the IPD meta-analyses, but there was no clear evidence for other types of interventions (table 3). There were no clear differences between the groups for overall or individual interventions for gestational diabetes defined by modified IADPSG criteria (table 3).

**Table 3:** Effects of lifestyle interventions on gestational diabetes defined by IADPSG and modified IADPSG criteria summarised using IPD alone and by supplementing IPD with study level data from studies that did not contribute IPD

Intervention and source	No of studies (No of women)	Odds ratio (95% CI)	95% PI	τ^2^ (95% CI)
**IADPSG criteria**				
Physical activity				
IPD only	3 (55)	0.92 (0.28 to 3.08)	0.03 to 32.47	0.00 (0.00 to 13.85)
IPD and aggregate data	5 (420)	0.93 (0.69 to 1.25)	0.66 to 1.31	0.00 (0.00 to 1.44)
Diet				
IPD only	2 (895)	0.71 (0.06 to 7.88)	Undefined	0.00 (0.00 to 14.9)
IPD and aggregate data	7 (2314)	0.81 (0.55 to 1.20)	0.35 to 1.90	0.08 (0 to 0.69)
Mixed				
IPD only	11 (5224)	0.89 (0.76 to 1.03)	0.76 to 1.04	0.00 (0.00 to 0.08)
IPD and aggregate data	17 (5892)	0.83 (0.71 to 0.96)	0.71 to 0.96	0.00 (0.00 to 0.12)
All				
IPD only	16 (6174)	0.86 (0.75 to 0.97)	0.75 to 0.97	0.00 (0.00 to 0.07)
IPD and aggregate data	29 (8626)	0.82 (0.72 to 0.93)	0.64 to 1.04	0.01 (0 to 0.11)
**Modified IADPSG criteria**				
Physical activity*				
IPD only	7 (1940)	0.88 (0.70 to 1.09)	0.70 to 1.10	0.00 (0.00 to 0.53)
Diet*				
IPD only	3 (1891)	0.64 (0.32 to 1.30)	0.08 to 5.11	0.00 (0.00 to 2.78)
Mixed				
IPD only	14 (9355)	1.08 (0.89 to 1.31)	0.68 to 1.72	0.04 (0.00 to 0.21)
IPD and aggregate data	15 (9622)	1.07 (0.89 to 1.29)	0.69 to 1.67	0.03 (0 to 0.19)
All				
IPD only	24 (13 186)	0.92 (0.78 to 1.10)	0.52 to 1.64	0.07 (0.02 to 0.23)
IPD and aggregate data	25 (13 453)	0.93 (0.79 to 1.09)	0.53 to 1.62	0.07 (0.02 to 0.21)

CI=confidence interval; IADPSG=International Association of Diabetes in Pregnancy Study Group; IPD=individual participant data; PI=prediction interval.

* No additional aggregate data studies available.

The contour enhanced funnel plots did not show clear evidence of asymmetry for the IPD meta-analysis of gestational diabetes defined by any criteria and by NICE criteria. The findings were consistent when non-IPD trials were added, and when high risk of bias IPD trials were excluded (supplementary web appendix 8).

### Differential effects of lifestyle interventions

We did not find a treatment-covariate interaction effect for maternal characteristics like body mass index, age, parity, and ethnicity in reducing gestational diabetes by any criteria. However, women with low educational levels were less likely to benefit than those with middle and high educational levels (low *v* middle interaction: odds ratio 0.68, 95% CI 0.51 to 0.90, τ^2^=0.00; low *v* high interaction: odds ratio 0.71, 95% CI 0.54 to 0.93, τ^2^=0.00). But the intervention was beneficial within all three educational level subgroups (table 4). No such differences were observed for gestational diabetes defined by NICE criteria (table 4). Our subgroup analyses by intervention components did not show differences in the effects by frequency, intensity, mode of delivery, timing, facilitator type, or setting. Interventions delivered in group formats (odds ratio 0.81, 95% CI 0.68 to 0.97; absolute risk reduction 2.5%, 95% CI 0.4% to 4.3%) and by newly trained providers (odds ratio 0.82, 95% CI 0.69 to 0.96; absolute risk reduction 2.4%, 95% CI 0.5% to 4.2%) showed greater benefits than individual formats and providers with previous training (supplementary web appendix 9).

**Table 4:** Treatment-covariate interaction estimates for lifestyle interventions on gestational diabetes defined by any criteria and NICE criteria in subgroups of pregnant women

Maternal characteristics	No of studies (No of women)	Treatment-covariate interaction		
		Interaction odds ratio (95% CI)	95% PI	τ^2^ (95% CI)
**Any criteria**				
Ethnicity: non-white *v* white	18 (8733)	0.98 (0.71 to 1.34)	0.71 to 1.34	0.00 (0.00 to 0.41)
Parity: multiparous *v* nulliparous	40 (19 574)	0.88 (0.75 to 1.03)	0.75 to 1.03	0.00 (0.00 to 0.17)
Education				
Middle *v* low	33 (10 887)	0.68 (0.51 to 0.90)	0.51 to 0.90	0.00 (0.00 to 0.49)
High *v* low	32 (10 794)	0.71 (0.54 to 0.93)	0.54 to 0.93	0.00 (0.00 to 0.41)
Age (years)				
≥20 *v* <20	24 (17 320)	1.00 (0.74 to 1.36)	0.74 to 1.36	0.00 (0.00 to 0.79)
Age (continuous)	52 (23 161)	1.00 (0.98 to 1.02)	0.98 to 1.02	0.00 (0.00 to 0.00)
Baseline body mass index				
Overweight *v* normal	33 (16 711)	0.98 (0.74 to 1.29)	0.50 to 1.90	0.09 (0.00 to 0.64)
Obese *v* normal	48 (21 080)	0.90 (0.72 to 1.11)	0.72 to 1.12	0.00 (0.00 to 0.37)
Body mass index (continuous)	54 (23 361)	1.00 (0.98 to 1.02)	0.98 to 1.02	0.00 (0.00 to 0.00)
**NICE criteria**				
Ethnicity: non-white *v* white	10 (5736)	0.73 (0.42 to 1.26)	0.34 to 1.57	0.05 (0.00 to 1.30)
Parity: multiparous *v* nulliparous	14 (9072)	0.98 (0.77 to 1.24)	0.77 to 1.25	0.00 (0.00 to 0.37)
Education				
Middle *v* low	8 (4312)	1.30 (0.78 to 2.15)	0.77 to 2.19	0.00 (0.00 to 1.12)
High *v* low	8 (4293)	1.15 (0.68 to 1.96)	0.67 to 1.99	0.00 (0.00 to 1.35)
Age (years)				
≥20 *v* <20	13 (10 461)	1.15 (0.87 to 1.51)	0.87 to 1.51	0.00 (0.00 to 0.73)
Age (continuous)	22 (11 990)	1.00 (0.99 to 1.02)	0.99 to 1.02	0.00 (0.00 to 0.00)
Baseline body mass index				
Overweight *v* normal	16 (7965)	1.11 (0.67 to 1.83)	0.34 to 3.56	0.23 (0.00 to 1.43)
Obese *v* normal	16 (9219)	1.07 (0.69 to 1.68)	0.40 to 2.87	0.17 (0.00 to 1.19)
Body mass index (continuous)	22 (9462)	1.00 (0.99 to 1.02)	0.99 to 1.02	0.00 (0.00 to 0.00)

CI=confidence interval; NICE=National Institute for Health and Care Excellence; PI=prediction interval.

### Effects on maternal and offspring outcomes

IPD meta-analyses of trials reporting gestational diabetes defined by any criteria did not provide clear evidence that lifestyle interventions reduce adverse pregnancy outcomes like hypertensive diseases, preterm birth, caesarean section, stillbirth, and small or large for gestational age babies. Among the types of interventions, physical activity based ones statistically significantly reduced the odds of caesarean section (odds ratio 0.83, 95% CI 0.72 to 0.96, τ^2^=0.00, 17 studies, 4527 women; absolute risk reduction 3.8%, 95% CI 0.9% to 6.4%), small for gestational age (odds ratio 0.72, 95% CI 0.56 to 0.92, τ^2^=0.00, 17 studies, 4594 women; absolute risk reduction 1.9%, 95% CI 0.5% to 3.0%), and large for gestational age babies (odds ratio 0.81, 95% CI 0.71 to 0.94, τ^2^=0.00, 17 studies, 4594 women; absolute risk reduction 2.7%, 95% CI 0.8% to 4.2%); no clear differences were observed for other outcomes. Diet based interventions reduced the odds of preterm birth (odds ratio 0.37, 95% CI 0.20 to 0.68, τ^2^=0.0, six studies, 1464 women; absolute risk reduction 6.6%, 95% CI 3.3% to 8.6%) compared with controls, with no clear reductions in other outcomes. No clear differences were observed for any maternal or offspring outcomes with mixed interventions (table 5).

**Table 5:** Effects of lifestyle interventions on pregnancy outcomes summarised using individual participant data alone

Outcome and intervention	No of studies (No of women)	Odds ratio (95% CI)	PI	τ^2^ (95% CI)
**Hypertensive disease**				
Physical activity	18 (4620)	0.87 (0.64 to 1.18)	0.42 to 1.79	0.09 (0.00 to 0.86)
Diet	8 (2980)	0.81 (0.55 to 1.17)	0.44 to 1.47	0.04 (0.00 to 0.73)
Mixed	28 (16 098)	1.10 (0.97 to 1.24)	0.97 to 1.24	0.00 (0.00 to 0.06)
All	54 (23 698)	1.03 (0.92 to 1.14)	0.92 to 1.14	0.00 (0.00 to 0.09)
**Preterm birth**				
Physical activity	15 (4504)	1.02 (0.78 to 1.34)	0.77 to 1.34	0.00 (0.00 to 0.29)
Diet	6 (1464)	0.37 (0.20 to 0.68)	0.19 to 0.71	0.00 (0.00 to 1.73)
Mixed	24 (14 801)	0.95 (0.79 to 1.13)	0.72 to 1.24	0.01 (0.00 to 0.13)
All	45 (20 769)	0.93 (0.80 to 1.07)	0.73 to 1.18	0.01 (0.00 to 0.11)
**Caesarean section**				
Physical activity	17 (4527)	0.83 (0.72 to 0.96)	0.72 to 0.96	0.00 (0.00 to 0.11)
Diet	8 (2829)	0.93 (0.78 to 1.11)	0.71 to 1.22	0.01 (0.00 to 0.38)
Mixed	24 (13 178)	0.99 (0.88 to 1.10)	0.71 to 1.37	0.02 (0.00 to 0.09)
All	49 (20 534)	0.93 (0.86 to 1.01)	0.73 to 1.19	0.01 (0.00 to 0.05)
**Stillbirth**				
Physical activity	7 (1218)	1.39 (0.86 to 2.25)	0.83 to 2.30	0.00 (0.00 to 3.57)
Diet	4 (1576)	0.65 (0.25 to 1.66)	0.18 to 2.32	0.00 (0.00 to 8.09)
Mixed	17 (7100)	0.75 (0.57 to 1.01)	0.56 to 1.01	0.00 (0.00 to 0.68)
All	28 (9894)	0.80 (0.64 to 1.01)	0.64 to 1.01	0.00 (0.00 to 0.42)
**Small for gestational age**				
Physical activity	17 (4594)	0.72 (0.56 to 0.92)	0.56 to 0.92	0.00 (0.00 to 0.27)
Diet	6 (1450)	0.89 (0.17 to 4.74)	0.02 to 48.57	1.65 (0.09 to 11.83)
Mixed	20 (11 470)	1.06 (0.94 to 1.20)	0.93 to 1.20	0.00 (0.00 to 0.08)
All	43 (17 514)	0.94 (0.82 to 1.09)	0.82 to 1.09	0.00 (0.00 to 0.18)
**Large for gestational age**				
Physical activity	17 (4594)	0.81 (0.71 to 0.94)	0.69 to 0.97	0.00 (0.00 to 0.11)
Diet	6 (1450)	0.72 (0.46 to 1.14)	0.44 to 1.18	0.00 (0.00 to 0.94)
Mixed	19 (11 236)	1.03 (0.92 to 1.16)	0.92 to 1.16	0.00 (0.00 to 0.05)
All	42 (17 280)	0.93 (0.85 to 1.02)	0.85 to 1.02	0.00 (0.00 to 0.05)

CI=confidence interval; PI=prediction interval.

### Network meta-analysis

**Fig 3:**
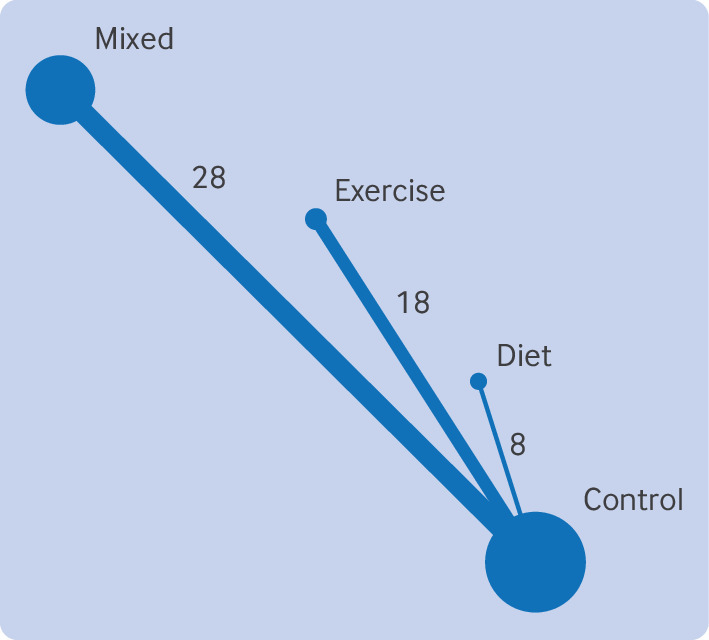
Network graph of included studies for gestational diabetes defined by any criteria, with thickness of lines and size of circles proportional to number of studies and number of women, respectively

A connected network was formed for gestational diabetes defined by any criteria, with minor heterogeneity between studies (τ=0.10; fig 3). Indirect intervention effects showed a reduction in the odds of gestational diabetes by 39% on average (odds ratio 0.61, 95% CI 0.46 to 0.83; absolute risk reduction 5.3%, 95% CI 2.2% to 7.5%) with physical activity based interventions compared with mixed interventions (table 6). Physical activity based interventions had the highest mean rank (1.1, 95% CI 1 to 2) and the highest probability of being ranked best intervention (89%), while mixed interventions had the lowest mean rank (3.8, 95% CI 3 to 4) and the highest probability of being ranked worst intervention (78.6%) (supplementary web appendix 10). We were unable to statistically test the consistency assumption owing to the geometry of the network.

**Table 6:** Network meta-analysis results for all possible comparisons summarised using individual participant data alone

Intervention	Control	Physical activity	Diet	Mixed
Control	—	1.55 (1.18 to 2.02)	1.23 (0.98 to 1.55)	0.95 (0.83 to 1.09)
Physical activity	0.65 (0.50 to 0.85)	—	0.80 (0.56 to 1.13)	0.61 (0.46 to 0.83)
Diet	0.81 (0.65 to 1.03)	1.26 (0.88 to 1.79)	—	0.77 (0.59 to 1.01)
Mixed	1.06 (0.92 to 1.21)	1.63 (1.21 to 2.20)	1.30 (0.99 to 1.71)	**—**

Values are odds ratio (95% confidence interval).

## Discussion

### Principal findings

The effects of lifestyle interventions in pregnancy on gestational diabetes vary by the diagnostic criteria used in clinical practice. The effects differed by maternal education and not by maternal body mass index, age, parity, or ethnicity. Although reductions in gestational diabetes were observed across all educational levels, the magnitude of the benefit was less in mothers with low education level. The effects were consistent across intervention characteristics, but benefits were greater when delivered in group formats and by newly trained providers. Physical activity based interventions appear to be the most effective among individual interventions. No differences were observed in maternal and perinatal outcomes in studies on overall lifestyle interventions reporting gestational diabetes. However, benefits were observed with individual interventions, such as reduction in caesarean section, and risks of small and large for gestational age babies with physical activity, and preterm birth with diet based interventions.

### Strengths and limitations

Our large IPD meta-analysis comprised randomised data for more than 24 000 women, resulting in enhanced precision and reliability of findings.^10^ By accessing the raw participant data, such as blood glucose levels, we were able to standardise the reported outcomes and assess the effects of interventions on gestational diabetes for various diagnostic criteria.^79^ Access to IPD also provided us with larger power to assess the differential intervention effects across various subgroups, which is not often possible in individual trials or in aggregate data. In addition to relative measures, we reported absolute risk reductions to help clinical interpretation, allowing clinicians and policy makers to better appreciate the potential public health impact of lifestyle interventions. We reported both confidence intervals and prediction intervals for transparency. Our primary interpretation was based on confidence intervals, in keeping with standard meta-analysis reporting conventions and the estimates typically used to guide clinical recommendations and policy.^80^ However, we also considered the prediction intervals in our interpretation, particularly in analyses with wider intervals that indicate potential variation in future settings. The interpretations based on confidence intervals and prediction intervals were similar across analyses, except for two comparisons: the overall effect of lifestyle interventions (IPD plus non-IPD trials) and physical activity based interventions (IPD alone) for gestational diabetes defined by any criteria. These differences should be taken into account when considering how the findings might translate to different clinical settings. By adding studies that did not contribute IPD to the IPD meta-analysis, we were able to provide the totality of evidence of the magnitude of effect of lifestyle interventions. Moreover, by undertaking sensitivity analyses by excluding lower quality studies, we were able to assess the consistency of the findings. The network meta-analysis allowed us to identify the most effective intervention to make decisions on the choice of interventions in practice.

Our work has limitations. Despite several attempts, we were unable to obtain IPD from many trials published up to March 2021. However, our IPD dataset accounted for 68% of all randomised participants across eligible studies. Included studies varied in the characteristics of participants and interventions, but through our subgroup analysis we were able to assess the differential effect in various populations and intervention components. Our network meta-analysis was limited by the absence of closed loops, which prevented formal assessment of consistency. Potential heterogeneity in intervention characteristics and differences in standard care may affect the assumption of transitivity in our network meta-analysis. However, the comparability of populations and our adjustment for key prognostic factors support the plausibility of the transitivity assumption. When examining continuous variables, we assumed linear trends, but further work might consider potential non-linear relationships for investigating treatment-covariate interactions.

The interventions varied in duration, intensity, timing, and provider, and we were only able to broadly define them as predominantly physical activity based, diet based, or mixed interventions. The mixed interventions group was heterogenous, with many trials having unstructured interventions. Not all individual trials systematically collected adherence or compliance data, so we were unable to assess the potential impact of intervention adherence on outcomes in our analyses. A third of trials that shared IPD did not report ethnicity in the data, and for those that did, the populations were mostly white. As a result, we were unable to explore the effects of ethnicity in detailed subcategories in the non-white group because of the wide variation in definitions of race and ethnicity in individual studies. We only reported the effects of lifestyle interventions on maternal and perinatal outcomes in studies that reported on gestational diabetes. The findings are likely to differ when all randomised trials on lifestyle interventions are included. Most trials were conducted in high income countries, limiting the generalisability of our findings to diverse global settings.

### Comparison with other studies

For overall lifestyle interventions, no clear evidence was found for a reduction in gestational diabetes across all diagnostic criteria. Although benefits were observed for IADPSG defined gestational diabetes, which has a relatively low threshold for diagnosis, the effects did not extend to NICE defined gestational diabetes. The findings also varied when non-IPD trials were added, and when low quality studies were excluded. In our interpretation of the findings, we considered the intervention to have an impact on an outcome if it consistently showed a benefit across all three analyses: IPD meta-analyses, including non-IPD trials, and excluding studies with high risk of bias. Among individual interventions, we found a consistent reduction in physical activity based interventions for gestational diabetes defined by any criteria in all three analyses.

Physical activity based interventions also ranked the highest among all three intervention types. The highly structured targeted approach of physical activity based interventions probably contributed to the observed large magnitude of effect.^81^ In our discussions with stakeholders, patient and public involvement and engagement groups highlighted that women usually stop all physical activity and exercise when found to be pregnant owing to concerns about the impact on pregnancy.^82^ In such a situation, any increase in physical activity is likely to show benefit. Our findings are similar to the observed benefits in preventing type 2 diabetes with physical activity in the general population.^83^ As in previously published reviews involving pregnant women, we did not observe a beneficial effect with the mixed approach.^10^ This could be because of the burden of simultaneous engagement across behaviour change interventions, which may affect adherence and compliance with the intervention in pregnancy.^81^ Systematic differences are likely between participants’ motivation and willingness to engage in the highly structured physical activity based trials and those with diet or mixed interventions.^84^

The benefits of lifestyle interventions were observed across educational levels, although the magnitude appeared smaller among women with low education. This observation suggests a potential social gradient in effectiveness.^85^ ^86^ We considered education to be a proxy for socioeconomic status.^87^ The reach, uptake, and adherence to lifestyle interventions are likely to be affected by barriers encountered by women from low socioeconomic backgrounds, including the perception of risk, previous negative experiences with lifestyle change, costs of healthy foods and access to gym facilities, neighbourhood safety to undertake physical activity, lack of access to e-health interventions, time constraints, and social pressures.^88^ ^89^ These factors may limit their ability to engage fully with interventions.

### Policy implications

Addressing maternal health inequities requires multilevel interventions that extend beyond individual behaviours to tackle the broader structural barriers and social inequities that shape health outcomes. Community based programmes that leverage existing social infrastructure and foster peer-to-peer support may be more successful in reaching marginalised populations and promoting sustainable lifestyle changes.^86^ Interventions designed with accessibility, cultural relevance, and support structures in mind may enhance engagement across educational groups. Understanding the behavioural, social, and structural determinants of adherence to the intervention is critical to advancing health equity. Although women from ethnic minority backgrounds are at high risk of gestational diabetes, we did not find variations in the effects of lifestyle interventions between white and non-white mothers. The findings are similar to the observed lack of differential effect of lifestyle interventions by ethnicity in preventing type 2 diabetes in the general population.^90^ We also did not find any variations in the effects of lifestyle interventions by maternal body mass index, age, or parity. Therefore, lifestyle interventions may benefit all women across maternal subgroups, irrespective of their baseline characteristics.

Although some characteristics of intervention delivery such as group based sessions and delivery by newly trained providers may enhance effectiveness, we found that lifestyle interventions offer benefit irrespective of how they are delivered. There is no one-size-fits-all approach, and the belief that a specific type, intensity, or format of lifestyle intervention is necessary to prevent gestational diabetes is not supported by our findings. While the size of benefit may vary, providing any form of lifestyle intervention is better than doing nothing. These findings support integrating lifestyle interventions into routine antenatal care as a practical and scalable strategy to improve outcomes.

The current focus in countries continues to be on early diagnosis and treatment of gestational diabetes.^91^ Practice level protocols and policy level guidance targeting pregnancy for preventing gestational diabetes are lacking. National programmes like the Diabetes Prevention Programme in the UK do not include the prevention of gestational diabetes.^92^ Clear policies are needed that highlight the benefits of lifestyle interventions in pregnancy. The conversations around lifestyle should be part of routine antenatal care. In particular, women should be reassured about the safety of physical activity interventions, and informed that any activity should be better than none. Access to green and blue spaces and financial support such as healthy start vouchers given in the UK will encourage women to improve their physical activity and diet.^93^ ^94^ Given the high prevalence of gestational diabetes and associated risks of short and long term complications in mothers and babies, even a small shift in the population distribution could have substantial public health benefits.

### Research implications

Future studies are needed on the barriers and facilitators at individual, interpersonal, community, organisational, and policy levels to help guide adaptations to optimise engagement and outcomes across diverse populations. Use of technology in delivery of lifestyle interventions may bring down the cost of delivering interventions at scale.^95^ A recent study found that women from lower socioeconomic groups found a specifically designed smart phone application helpful in their engagement with a dietary and physical activity intervention.^96^ However, the effectiveness and acceptability of technology enabled solutions will need to be rigorously assessed once developed and deployed.^97^ Disaggregated ethnicity data should be collected and reported in individual studies to better explore generalisability of findings and ensure interventions do not widen the inequality gap.^98^ ^99^

Future studies could also examine duration of follow-up as a potential effect modifier, which we were unable to assess in our prespecified analyses. Well designed follow-up studies are needed to assess the long term impact of lifestyle interventions in pregnancy on the metabolic health of mothers and their babies. A pressing need exists for high quality trials in low and middle income countries where the burden of gestational diabetes is rapidly rising but resources for intervention may be limited.^100^ Future research should focus on implementation science approaches to inform translation of these findings into equitable, culturally appropriate, and scalable interventions embedded within supportive health systems and policy environments.

### Conclusion

Lifestyle interventions in pregnancy are likely to prevent gestational diabetes, with effects varying by diagnostic criteria used. Benefits were smaller among women with lower education, highlighting equity gaps. Interventions delivered in group formats and by newly trained providers enhanced effectiveness. Physical activity based interventions were most effective. Implementation strategies should aim to prioritise equitable access and optimise delivery to maximise impact.

**What is already known on this topic**

Lifestyle interventions such as physical activity and diet prevent type 2 diabetes in the general population and have the potential to prevent gestational diabetes in pregnancy
Physical activity and diet based interventions in pregnancy are effective in reducing gestational weight gain, but evidence varies about their effects on gestational diabetes, or which intervention is most effective
Studies are needed analysing whether the effects of lifestyle intervention vary in different subgroups of women according to their body mass index, age, parity, ethnicity, and socioeconomic status, or by intervention characteristics

**What this study adds**

The global i-WIP Collaborative Group conducted a large individual participant data (IPD) meta-analysis and showed that lifestyle interventions prevent gestational diabetes, with effects varying by diagnostic criteria
The effects of lifestyle interventions on gestational diabetes did not vary across maternal characteristics like body mass index, age, parity and ethnicity, but varied by educational levels, where women with low education levels benefitted less
The effects were similar irrespective of frequency, intensity, facilitator type, setting, mode of delivery, and timing of interventions; greater benefits were observed with group formats and newly trained providers, and physical activity based interventions were consistently most effective

## Data Availability

All data requests should be submitted to the corresponding author. Access to available anonymised data may be granted after review and appropriate agreements being in place.

## Web extra

Extra material supplied by authors

Web appendix: Supplementary web appendices

## Notes

i-WIP Collaborative Group: Kelly Allison, Ellen Althuizen, Alka Kothari, Carla Assaf-Balut, Arne Astrup, Erica Baciuk, Ruben Barakat, Isabelle Marc, Annick Bogaerts, Jose Guilherme Cecatti, José F Cordero, Gustaaf Dekker, Roland Devlieger, Jodie M Dodd, Nermean El Beltagy, Fabio Faccchinetti, María Luisa Garmendia, Kirsti Krohn Garnæs, Nina RW Geiker, Kym Guelfi, Isabelle Guelinckx, Lene AH Haakstad, Cheryce Harrison, Hans Hauner, Marquis Hawkins, Amy Hui, Kirby Jeffries, Dorte M Jensen, Maria Kennelly, Janette Khoury, Julia Kunath, Riitta Luoto, Elizabeth McCarthy, Fionnuala McAuliffe, Narges Sadat Motahari-Tabari, Siv Mørkved, Fernanda Surita, Simony Lira do Nascimento, Carrie Nobles, Chrsitne M Olson, Ming Jing Ong, Nicolette Oostdam, Mireia Peleaz, María Perales, Elisabetta Petrella, Suzanne Phelan, Lucilla Poston, Julie Owens, Kathrin Rauh, Kristina Renault, Kristiina Rono, Jonatan Ruiz, Linda R Sagedal, Kjell Å Salvesen, Tânia T Scudeller, Alexis Shub, Garry X Shen, Signe N Stafne, Tarja I Kinnunen, Helena Teede, Serena Tonstad, Mireille van Poppel, Christina Vinter, Ingvild Vistad, Jennifer Walsh, Jane Willcox, S Wolff, Seonae Yeo.

Contributors: JA, DC, JZ, TR, NH, GAH, SAS, KN, LP, APB, NM, RM, SI, FA, HT, RDR, and ST developed the protocol. DC, AB, VK, MZ, HM, MBK, and GM carried out the literature search, study selection, and data extraction. DC and JA led the project, acquired the IPD, mapped the variables, cleaned and quality checked the data. DC, JE, GRC, COO, MM, TR, JZ, and RDR conducted data analysis. LP, HT, CH, KJG, ST, SAY, JGC, FF, SMT, KMR, DMJ, ALCP, FMM, HH, RB, NRWG, CAV, SP, TIK, and AK contributed data and provided input at all stages of the project. JA and ST wrote the initial report draft. All authors critically appraised the final draft of the report. The corresponding author attests that all listed authors meet authorship criteria and that no others meeting the criteria have been omitted. JA is the guarantor.

Transparency: The lead author (the manuscript’s guarantor) affirms that the manuscript is an honest, accurate, and transparent account of the study being reported; that no important aspects of the study have been omitted; and that any discrepancies from the study as planned (and, if relevant, registered) have been explained.

Dissemination to participants and related patient and public communities: Findings were disseminated to key stakeholders including patients and members of the public at a workshop event. We will work with our patient and public involvement group to develop an animated lay summary of the study findings. A written lay summary has also been prepared for the funder and is provided in the supplementary material.

Provenance and peer review: Not commissioned; externally peer reviewed.
