# Supplementary material for: Effects of lifestyle interventions in pregnancy on gestational diabetes: individual participant data and network meta-analysis
Source: BMJ. 2026 Jan 7;392:e084159. doi: 10.1136/bmj-2025-084159 (PMC12771886; doi:10.1136/bmj-2025-084159)
Supplement: Supplementary file 1 — Web appendix: Supplementary web appendices [file allj084159.ww.pdf]

Appendix 1 Search strategy for identification of randomised trials on lifestyle interventions in pregnancy and maternal and offspring outcomes

Search strategy for Medline via Ovid

Item	Term
1	Pregnancy/
2	pregnan*.tw.
3	Gravidity/
4	gravid*.tw.
5	gestation*.tw.
6	Pregnant Women/
7	pregnant wom#n.tw.
8	(child adj3 bearing).tw.
9	childbearing.tw.
10	matern*.tw.
11	or/1-10
12	Weight Gain/ph [Physiology]
13	weight gain*.tw.
14	Weight Loss/ph [Physiology]
15	weight loss*.tw.
16	weight change*.tw.
17	Obesity/dh, me, ph, pc, px, th [Diet Therapy, Metabolism, Physiology, Prevention & Control, Psychology, Therapy]
18	obes*.tw.
19	Adiposity/ph [Physiology]
20	adipos*.tw.
21	Overweight/dh, me, ph, pc, px, th [Diet Therapy, Metabolism, Physiology, Prevention & Control, Psychology, Therapy]
22	overweight*.tw.
23	Body Mass Index/
24	bmi.tw.
25	or/12-24
26	exp Randomized Controlled Trial/
27	"randomized controlled trial".pt.
28	"controlled clinical trial".pt.
29	(random$ or placebo$).tw,sh.
30	((singl$ or double$ or triple$ or treble$) and (blind$ or mask$)).tw,sh.
31	single-blind method/
32	double-blind method/
33	or/26-32
34	11 and 25 and 33
35	exp Animals/
36	(rat$ or mouse or mice or hamster$ or animal$ or dog$ or cat$ or bovine or sheep or lamb$).af.
37	35 or 36
38	Humans/
39	human$.tw,ot,kf.
40	37 or 38
41	37 not (37 and 40)
42	34 not 41

Appendix 2: Outcome definitions for the individual participant data (IPD) meta-analysis of lifestyle interventions on gestational diabetes

**Primary outcomes**	• Gestational diabetes defined within the study by any established criteria<br>• Gestational diabetes defined as per 2015 NICE criteria (fasting glucose 5.6 mmol/L or above, or 2-hour glucose 7.8 mmol/L or above after a 75 g oral glucose tolerance test)
**Secondary outcomes**	• Gestational diabetes as defined using the IADPSG criteria (fasting glucose 5.1 mmol/L or above, or 1-hour glucose 10 mmol/L or above after a 75 g oral glucose tolerance test, or 2-hour glucose 8.5 mmol/L or above after a 75 g oral glucose tolerance test)<br>• Gestational diabetes as defined using the modified IADPSG criteria (fasting glucose 5.1 mmol/L or above, or 2-hour glucose 8.5 mmol/L or above after a 75 g oral glucose tolerance test)<br>• ***Maternal:*** hypertensive diseases including pre-eclampsia, preterm birth, caesarean section, preterm birth and need for pharmacological therapy for hyperglycaemia<br>• ***Offspring:*** shoulder dystocia, respiratory distress syndrome, neonatal hypoglycaemia, stillbirth, neonatal death, perinatal death, Apgar score at 1 and 5 min, birthweight, gestational age at birth, small/large for gestational age and admission to the neonatal unit

iWIP GDM

Statistical Analysis Plan

Draft Version 1.0
08 March 2022

Person(s) contributing to the analysis plan		
Name(s) and position(s)	Richard Riley<br>Joie Ensor<br>Dyuti Coomar<br>Shakila Thangaratinam<br>John Allotey<br>Anneke Damen	Senior study statistician<br>Study statistician<br>Study coordinator<br>PI<br>Co-lead<br>Independent Statistician
Authorisation		
Position	**Chief or principal investigator**	
Name	**Shakila Thangaratinam**	
Signature	[illegible]	
Date		
Position	**Senior study statistician**	**Study statistician**
Name	**Richard Riley**	**Dyuti Coomar**
Signature	[illegible]	Dyuti Coomar
Date		
Position	**Independent statistician**	
Name	**Anneke Damen**	
Date reviewed	28 July 2022	

This document was created based on the iWIP analysis plan (Approved version 1.0; 14/07/2015) developed by Dyuti Coomar and Richard Riley.

1 INTRODUCTION

1.1 Purpose of statistical analysis plan

The purpose of this document is to provide details of the statistical analyses and presentation of results to be reported within the principal paper(s) of the iWIP study. Subsequent papers of a more exploratory nature (including those involving baseline data only) will not be bound by this strategy but will be expected to follow the broad principles laid down in it. Any exploratory, post-hoc or unplanned analyses will be clearly identified in the respective study analysis report.

The structure and content of this document provides sufficient detail to meet the requirements identified by the International Conference on Harmonisation (ICH).

The following guidelines were reviewed in preparation for writing this document:

- Study grant application to NIHR HTA (Award ID: NIHR129715)
- Published study protocol[1]
- ICH E3 Structure and content of clinical study reports
- Reporting guidelines PRISMA-IPD[2]
- The textbook "Individual Participant Data Meta-Analysis: A Handbook for Healthcare Research"[3]

1.2 Members of the writing committee

Dyuti Coomar, Richard Riley and Joie Ensor were primarily responsible for writing the Statistical Analysis Strategy and with Dyuti Coomar responsible for writing the computer code implementing the analysis strategy and implementing the strategy at the point of analysis. If decisions are required during the analysis, they will be discussed with an independent statistician (Anneke Damen).

This document has been developed prior to examination of trial data and will not be implemented prior to final approval.

1.3 Summary

The full analysis plan outlined in this document was developed prior to database lock. The plan covers the analyses required to meet all primary and secondary objectives. In the event of a discrepancy, the analyses described in the Statistical Analysis Plan will supersede those outlined in the grant application and protocol.

1.4 Changes from planned analysis in the published protocol

Primary objective:

- None

Secondary objectives (see section 1):

- Aggregate data from non-IPD studies will not be added to IPD studies for Secondary Objective 1 due to lack of data.

2 STUDY OBJECTIVES AND ENDPOINTS

2.1 Study objectives

2.1.1 Primary objectives

1. To evaluate the effects of diet and physical activity-based lifestyle interventions in pregnancy, across all interventions, and for each type of intervention (diet-based, physical activity-based and mixed) on gestational diabetes as defined by the National Institute for Health and Care Excellence (NICE)[4] and by study authors.

2. To assess the differential effects of interventions according to the maternal characteristics (BMI, age, parity, ethnicity and socioeconomic status) on gestational diabetes.

2.1.2 Secondary objectives

1. To evaluate the effects of the interventions on critically important (1) maternal complications such as hypertensive diseases, caesarean section or preterm birth; and (2) offspring complications such as stillbirth, large for gestational age or admission to the neonatal unit in women with gestational diabetes.
2. To categorise the interventions by core components and to undertake network meta-analysis to rank them by effectiveness.
3. To assess the effects of interventions for specific other definitions of gestational diabetes (WHO[5], International Association of Diabetes and Pregnancy Study Group (IADPSG)[6], modified IADPSG[7], [8], and American Diabetes Association (ADA)[9]), and on fasting and 2-hour post-prandial glucose levels.
4. To determine the cost-effectiveness of interventions using decision–analytical modelling

2.2 Outcome measures

2.2.1 Primary outcomes

1. Gestational diabetes mellitus (GDM) defined as per
   i. National Institute for Health and Care Excellence (NICE) 2015 criteria: fasting glucose 5.6 mmol/L or above, and 2- hour glucose 7.8 mmol/L or above after a 75 g oral glucose tolerance test or
   ii. GDM as defined within the study by established criteria
2. Differential effects of interventions across subgroups based on maternal BMI, age, parity, ethnicity and socioeconomic status

2.2.2 Secondary outcomes

1. Maternal and /or offspring complications in women with GDM (The outcomes marked in bold are the ones we are most interested in)

   Maternal complications: **hypertensive diseases including pre- eclampsia, caesarean section, preterm birth** and need for pharmacological therapy for hyperglycaemia

   Offspring complications: shoulder dystocia, respiratory distress syndrome, neonatal hypoglycaemia, stillbirth, neonatal death, perinatal death, Apgar score at 1 and 5 min, birth weight, gestational age at birth, **small/large for gestational age and admission to the neonatal unit**
2. Categorisation of the interventions by core components and undertake network meta-analysis to rank them by effectiveness
3. Gestational diabetes as defined specifically using the following definitions:
   i. International Association of Diabetes in Pregnancy Study Group (IADPSG): fasting glucose 5.1 mmol/L or above, or 1- hour glucose 10.0 mmol/L or above, or 2- hour glucose 8.5 mmol/L or above after a 75 g oral glucose tolerance test
   ii. modified IADPSG: fasting glucose 5.1 mmol/L or above, or 2- hour glucose 8.5 mmol/L or above after a 75 g oral glucose tolerance test
   iii. World Health Organisation (WHO): fasting glucose 5.1–6.9 mmol/l, or 1-h glucose 10.0 mmol/l, or 2-h glucose 8.5–11.0 mmol/l following a 75 g oral glucose load

iv. American Diabetes Association (ADA): (One-step strategy)

Perform a 75-g OGTT, with plasma glucose measurement when patient is fasting and at 1 and 2 h, at 24–28 weeks of gestation in women not previously diagnosed with diabetes.

The OGTT should be performed in the morning after an overnight fast of at least 8 h.

The diagnosis of GDM is made when any of the following plasma glucose values are met or exceeded:

- Fasting: 92 mg/dL (5.1 mmol/L)
- 1 h: 180 mg/dL (10.0 mmol/L)
- 2 h: 153 mg/dL (8.5 mmol/L)

4. Cost- effectiveness of interventions using decision–analytical modelling

GDM will be calculated for each of the definitions using the oral glucose tolerance test (OGTT) data provided in the IPD. If a study provides OGTT data for multiple points (<24 weeks, 24-30 weeks, >30 weeks), a patient will be considered to have GDM if the patient satisfies the criterion at any timepoint. If a study provides only dichotomized GDM information, it will be included in the primary outcome. If we know which GDM definition they used, then we can further include it in the secondary objective (for that particular definition).

3 STUDY METHODS

3.1 Overall study design and plan

Study Design: Individual participant data (IPD) meta-analysis of randomised trials.

Interventions: These vary across trials but can be categorised broadly as either diet-related, exercise-related, or a mixed approach of the two.

Control: Standard antenatal care as per local practice.

3.2 Identifying Studies

We will update the literature search using our existing search strategy[10] to identify new trials that have been published since the completion of our previous review[11]. We will search MEDLINE, Embase, BIOSIS, LILACS, Pascal, Science Citation Index, Cochrane Database of Systematic Reviews, Cochrane Central Register of Controlled Trials, Database of Abstracts of Reviews of Effects and Health Technology Assessment Database without language restrictions up to 28th March 2022. Two independent reviewers will extract data in duplicate.

3.3 Inclusion and exclusion criteria

Trials with random allocation (individual or cluster) on diet and physical activity-based interventions in pregnancy compared with standard antenatal care will be eligible for inclusion.

Any trials that included women at baseline with gestational diabetes will be excluded. We will only include pregnant women with a BMI of ≥18.5 kg/m2 in early pregnancy to exclude women who are underweight. We will evaluate three main interventions for gestational diabetes prevention in pregnancy: diet-based, physical activity-based, and mixed approach interventions incorporating diet and physical activity components underpinned by behavioural approach. Diet-based interventions include various dietary patterns such as Mediterranean-style diet, low calorie diet and low glycaemic index diet, which are offered by clinicians, dietitians, physiotherapists, or commercial companies in both primary and secondary care settings. The interventions are delivered using vehicles such as print or digital media, phone, face-to-face meetings in either one-on-one or group sessions, and are commenced at various time points in pregnancy and delivered in an intense regimented or pragmatic

manner. The physical activity-based interventions involved moderate exercise such as dance-based exercise programmes, water-based physical activity, stationery cycling, light intensity resistance training or enhanced routine daily activity, including walking. The mixed approach includes both diet and physical activity. Studies assessing other weight loss interventions such as pharmacotherapy or surgery will not be included.

We have previously identified the various interventions for inclusion in the IPD meta-analysis[12], [13]. We will map the components and taxonomy of the interventions against the clinical outcomes.

3.4 Sample size determination

Although no formal sample size requirements are necessary for meta-analysis (as you are synthesising the existing evidence), it is helpful to consider the potential power of our IPD meta-analysis in comparison to single trials in this field.

Brookes et al note that about four times the size of a single trial is required to detect an interaction with the same size as the overall treatment effect[14]. We currently have access to the shared data for 24 766 women (58 studies). The sample size has the potential to be increased further to 27 538 women (71 studies) and beyond, depending on decisions to share data by other identified studies, and by the number of new studies published up to March 2021. We have undertaken a simulation-based approach to calculate the power of estimating genuine treatment–covariate interactions in our planned IPD meta-analysis, conditional on the number of trials, number of participants available in each trial and the covariate characteristics (eg, proportion of Caucasian subjects, mean and SD of BMI) while allowing for between-study heterogeneity in the treatment effect and the control group risk[15], [16]. We calculated the power to detect a particular treatment–covariate interaction effect size in the subset of trials that report each covariate of interest (BMI, age, ethnicity, parity and socioeconomic status). Table 2 shows the power estimates, assuming an interaction between covariate and treatment effect corresponding to 30% (OR 0.70) and 25% reductions in gestational diabetes (OR 0.75) across covariate (sub)groups, using the sample size of available IPD (58 trials), and also if all 71 trials shared their IPD. We expect to have sufficient events for the overall effect, and for most of the secondary outcomes with 5291 caesarean sections, 1154 preterm births; 1769 hypertensive diseases; 2483 large-for-gestational age babies; and 1420 babies admitted to the neonatal unit.

Table 2
Estimated power by simulation based on the IPD currently available in the i-WIP database*

Covariate (subgroup) of interest		Assuming all 71 trials identified so far provide their IPD		For the 58 trials with IPD available/agreed	
		Trials (total participants) available for the covariate (n)	Estimated power by simulation (%)	Trials (total participants) available for the covariate (n)	Estimated power by simulation (%)
Assuming an interaction between covariate and treatment effect that corresponds to an OR of 0.70					
BMI	Obese versus non-obese	70 (27 722)	99.0	56 (24 443)	97.6
Age	Continuous assuming linear trend	69 (27 422)	79.1	55 (24 143)	78.4
Ethnicity	Caucasian versus non-Caucasian	48 (21 958)	93.4	39 (19 394)	90.4

Covariate (subgroup) of interest		Assuming all 71 trials identified so far provide their IPD		For the 58 trials with IPD available/agreed	
		Trials (total participants) available for the covariate (n)	Estimated power by simulation (%)	Trials (total participants) available for the covariate (n)	Estimated power by simulation (%)
Parity	Nulliparous versus multiparous	59 (22 253)	95.4	48 (19 718)	93.4
Socioeconomic status	High versus low	50 (21 136)	95.0	41 (19 426)	90.0
Assuming an interaction between covariate and treatment effect that corresponds to an OR of 0.75					
BMI	Obese versus non-obese	70 (27 722)	92.8	56 (24 443)	89.2
Age	Continuous assuming linear trend	69 (27 422)	72.4	55 (24 143)	65.2
Ethnicity	Caucasian versus non-Caucasian	48 (21 958)	79.8	39 (19 394)	74.4
Parity	Nulliparous versus multiparous	59 (22 253)	85.8	48 (19 718)	77.2
Socioeconomic status	High versus low	50 (21 136)	85.4	41 (19 426)	83.2

*Assuming baseline risk of gestational diabetes is 11% on average, varying from 2% to 43% according to trial characteristics.

BMI, Body Mass Index; IPD, individual participant data; i-WIP, International Weight Management in Pregnancy.

4 DATA COLLECTION

4.1 Collection of Aggregate Data

We will extract relevant study data, including population, intervention(s), comparison(s) and outcome information (measure and timing) for each study. For studies identified till March 2021, we will contact primary study authors for additional information when necessary. For studies identified from March 2021 to March 2022, we will not request IPD due to time constraints. We will only extract aggregate data. We will assess potential risk of bias for each study meeting selection criteria.

4.2 Collection of Individual Participant Data

The original individual participant data will be requested from the authors. We will identify contact information for study authors from PubMed or from the Internet and will email authors listed as 'contact authors' to tell them about our IPD meta-analysis and to ask if they are willing to share their trial data. If there is no response from the contact author, another investigator from the study will be contacted. We will contact each study at least three times.

We will contact participating study authors to provide additional information about the study and how to send us their IPD. Methods for receiving raw data from investigators may vary depending on the security concerns of their individual institutions; however, data may be obtained by e-mail, or by a secure transfer system. After data have been received, they will be stored on the web-based server at CREP (see section 5.3.1). We will accept databases in all formats in order to minimize the amount of work for primary study authors, however ideally the format will be a two-dimensional spreadsheet format with one subject per row, and variables listed in columns. Each raw dataset will be saved in their original format and then converted to a common format.

5 GENERAL ISSUES FOR STATISTICAL ANALYSIS

5.1 Blinding of the statistical analysis

Due to the nature of the study blinding is not practical. Study results as published will be compared against analysis performed which would automatically unblind.

5.2 Analysis populations

All participants will be analysed as they were allocated. Initially we will perform complete case analysis. However, if baseline imbalance is observed we will consider imputing missing data to analyse the full sample. Section **Error! Reference source not found.** describes which women will be excluded from analysis.

5.3 Database

5.3.1 Description

Original datasets for the iWIP project are uploaded and stored using the CREP (Centro Rosarino de Estudios Perinatales, Rosario, Argentina) web-based server. CREP is a WHO Collaborative Centre in Child and Maternal Health. The existing i-WIP database will be expanded to include new variables like OGTT values, maternal and neonatal complications, which were not included previously.

Unless otherwise specified all data manipulations are performed and documented within this environment. We will follow the established, tried and tested procedures used for data harmonization in the i-WIP database[17]. This will facilitate smooth and timely execution of the most time-consuming stage of the study, that is, data cleaning and its harmonization. The meta-dataset will then be transferred for final data checks and analysis.

5.3.2 Data Quality

Datasets included in the iWIP analysis are expected to be clean upon receipt from the trial team. Range checks on the variables used during the analysis will be performed. A re-analysis of the results published by the owner of the individual datasets will be limited as not all data used during their analysis might be provided. However, we will analyze each study individually part of the two step meta-analysis.

5.3.3 Database freeze

The statistician responsible for the analysis will conduct or oversee additional data checks. These include things such as range checks, logical and consistency checks which may not be picked up by checks performed at the individual level. This will be performed before uploading the individual datasets onto the webserver and before merging the individual datasets into the meta dataset.

Datasets will only be included in the study if they are with the study coordinator before end August 2021. Thereafter no further datasets will be added. All data manipulations will be performed with the CREP web-based server environment by the end of February 2022. If during the analysis any

unforeseen queries are generated, they will be dealt with on a case-by-case basis. Any subsequent changes made to the data will be recorded and reported.

5.4 Analysis software

The analysis will be carried out using Stata version 16 and R software.

5.5 Methods for missing Data

We will assess and summarize participant-level missing variable and outcome data. Individual participants within each trial with missing outcome data will generally be excluded from that specific analysis, though we will check that there is not a systematic difference in the amount of missing outcomes per group. We will adjust for key prognostic factors (e.g. age) in the analysis. If a covariate is missing, we will use the mean age in the study (continuous covariates) or the missing indicator method (for categorical covariates), which is more efficient than multiple imputation and appropriate for randomised trials.[18]

5.6 Method for handling centre and cluster effects within each trial

In cluster randomized trials, the clustering will be accounted for using random effects. For individual randomized studies no adjustment for center will be made.

5.7 Method for handling clustering of participants within trials in the meta-analysis

In the one-stage meta-analysis, clustering of participants within trials will be accounted for by including a separate intercept term per trial. In the two-stage meta-analysis, the clustering of participants within trials is accounted for by analyzing each trial separately in the first stage.

5.8 Method for handling randomisation stratification factors

Stratification or minimization factors used in the randomization of each study will not be adjusted for in the analysis.

5.9 Method for selecting other variables that will be adjusted for

We will aim to produce conditional treatment effects, that account for the prognostic factors of age and BMI. These are expected to be available in all studies. In clustered trials, centre effects (as described in 5.6) will also be included.

5.10 Derived and computed variables

Patient characteristics are recorded in various formats within the individual datasets. The meta-dataset format of each characteristic will be chosen using the least common denominator but also considering the number of studies which uses a certain format and the sample size of these studies. We will aim to balance loss of information through dichotomizing with the number of studies that can be included using a certain format. All derived and computed variables will be documented in the SQL scripts and Stata do-files.

All variables will be converted to the same scale for all trials.

Baseline activity was recorded in a variety of ways such as sedentary/active or using Met-min/wk, using various different questionnaires. It was not dichotomized into a binary measurement of sedentary vs at least somewhat active nor harmonized over the different studies due to lack of clarity on how to do it.

Outcome measurements of small and large for gestation age will be defined as less than 10th or more 90th centile respectively. Centile of birthweight will be calculated using the calculator provided on https://www.omnicalculator.com/health/birthweight-percentile.

5.11 Dealing with multiple arms

If trials have two or more intervention arms, then these will be accounted appropriately in the meta-analysis. If the multiple arms relate to different intervention types (for example, one is exercise-related and one is diet-related), and a usual care arm is available, then each intervention arm will be considered separately, to estimate effects (i.e. intervention or interaction effects) for each intervention in relation to usual care. These can then be included in subsequent meta-analyses for each intervention type separately. If a trial has multiple interventions of the same type (e.g. two exercise-related interventions),

then the average effect of these two interventions will be used in subsequent meta-analyses for that intervention type.

6 DESCRIPTIVE ANALYSES

We will describe study-level and participant-level characteristics of included studies. We will compare study-level characteristics and aggregate data from studies participating in the IPD analysis with those from studies that are eligible but do not supply data to the collaborative. Essentially, we will examine if the IPD studies available are a representative (unbiased) sample of the full set of existing studies, as recommended by Ahmed et al [19].

Appendix B and C show template tables for presentation of descriptive, main and economic analyses.

6.1 Baseline comparability of randomised groups

6.1.1 Demographics

- Maternal Age
- Height
- Ethnicity
- Education
- Smoking
- Socioeconomic status

6.1.2 Obstetric history

- Gravida
- Parity

6.1.3 Baseline weight

- Baseline Obesity (>30 BMI)

6.1.4 Baseline medical exam

- Pre-existing diabetes
- Pre-existing hypertension
- Pre-existing PIH (pregnancy induced hypertension)

6.2 Comparison of compliance to treatment and protocol

Some data will be available on adherence to intervention. If this is deemed sufficient a sensitivity analysis will be performed.

6.3 Comparison of study characteristics

For each study separately we will present baseline characteristics. Additionally, we will compare study information as available.

Study information:

- Country
- Year of publication
- Sample size
- Randomisation (cluster/individual)
- Risk of bias overall as defined in section 7.3.2 (low risk, and medium or high risk)

7 ANALYSIS OF PRIMARY OUTCOME

7.1 Definition of outcome measure

All definitions for outcomes have been provided in Section 2.2

7.2 Primary analysis

All analysis will be on an intention-to-treat principle and all estimates of effect size will be presented as point estimates, with corresponding 95% confidence intervals and p-values.

7.2.1 Method of meta-analysis

The effectiveness of the diet and physical activity-based interventions will be assessed using IPD meta-analytical framework[20]. GDM as defined by NICE [4] or GDM as defined within the study by established criteria will be the main outcome. For each intervention type (all interventions, diet-based, physical activity-based and mixed approach), we will perform one-stage and two-stage IPD random-effect meta-analyses to obtain the pooled (summary) intervention effect on GDM using restricted maximum likelihood (ML) estimation. As GDM is a binary outcome, the two-stage approach will fit a logistic regression in the first stage to obtain intervention effect estimates, and these will be pooled in the second stage using a random-effects meta-analysis. One-stage models will use a logistic regression random-effects framework, with intercepts stratified by study to account for clustering, and covariates centred to improve estimation[21]. CIs will be inflated to account for uncertainty in variance estimates (eg, using Hartung-Knapp and Kenward-Roger corrections for two-stage and one-stage approaches, respectively) [22]. All approaches will adjust for a few key prognostic factors (described in section 5.9) available in all the IPD studies, to ensure conditional treatment effects are summarised. Adjustment factors will be stratified by study and, in one-stage analyses, will be centered to improve estimation.

One-stage and two-stage analyses usually give similar results unless events are sparse, and so any discrepancies will be resolved[23]. We will use a random-effects meta-analysis approach, which allows for between-study heterogeneity in intervention effect, which is anticipated. Heterogeneity will be summarised using the I-squared statistic (which provides the proportion of total variability that is due to between-study heterogeneity) and the estimated between-study variance ('tau2'). To reveal the impact of heterogeneity more clearly, if there are more than 5 studies, we will also calculate a 95% prediction interval for the intervention effect when applied in an individual clinical setting. The aforementioned analyses will also be undertaken for secondary outcomes, and GDM will be defined using other specific criteria such as IADPSG, modified IADPSG, WHO and ADA.

For the binary outcomes of GDM, neonatal or maternal complications, the binomial nature will be suitably modelled using a logistic regression in each trial separately with intervention as a covariate. For any continuous neonatal outcomes, we will use analysis of covariance in each trial to regress the final outcome value against the intervention.

7.2.2 Intervention effect modifiers

When examining intervention effect modifiers, models will be extended to include interaction terms between participant-level covariates and the intervention. In the two-stage approach, the interaction estimates are obtained in the first stage and then pooled in a random effects meta-analysis model in the second stage. In the one-stage approach, the interaction term is included with all other terms stratified by trial (to avoid aggregation bias), with centering to improve estimation.

We will consider the following baseline characteristics as effect modifiers:

- Baseline BMI in kg/m^2
- Age in years
- Ethnicity, defined as caucasian vs non-caucasian
- Parity, defined as nulliparous vs multiparous
- Baseline medical conditions, defined as none vs at least one of baseline diabetes mellitus or hypertension.

For the interactions, continuous covariates (BMI and age), will be analysed on their continuous scale, rather than categorization[24]. However, to translate the results clinically, after the analysis we will report the effect of the covariate-treatment interaction on the intervention effect at a range of covariate values, for example using a graphical display.

To examine non-linear treatment-covariate relationships for continuous covariates, we will use a two-stage multivariate random effects meta-analysis. In the first stage, a restricted cubic spline with 3-4 knots will be fitted in each study separately (with the same knot positions in each), and the parameters of this interaction with treatment will be synthesized in the second stage using a multivariate meta-analysis, accounting for the within-study and between-study correlation between parameters.

Subgroup analyses, if not carefully planned, can lead to misleading results e.g., due to the play of chance with multiple testing[25]**.** Thus, caution will be used in interpretation of the collective set of subgroup results, and adjustment for multiple testing considered as necessary

7.3 Other analysis supporting the primary (incl. sensitivity analyses)

The sensitivity analysis will explore sources of bias. We will explore the potential for, and possible impact of, both publication bias and unavailable data, according to recent guidelines[19].

7.3.1 Sources of bias: small study effects and publication bias

For each analysis containing 10 or more studies small study effect (potential publication bias) will be investigated through the construction of contour-enhanced funnel plots and appropriate statistical tests for funnel plot asymmetry (Egger's test for continuous outcomes and Peter's test for binary outcomes); that is, the tendency for smaller studies to provide more positive findings. We will recognise that, especially where heterogeneity exists, publication bias may be one of several reasons for any small study effects identified. The restriction of 10 studies is due to the low power of identifying small study effects with few studies[26].

7.3.2 Sources of bias: study quality

We will use the risk of bias tool developed by the Cochrane Collaboration to score the quality of (the IPD from) each study. [29] This will be based on study characteristics and supplemented with information from IPD when provided. Sensitivity analyses will examine the robustness of meta-analysis results to the exclusion of studies with increased risk of bias. Studies will be excluded in a sensitivity analysis if none of the risk of bias items is low or the risk in at least one of the following items is high:

- Risk of bias: Randomisation (method)
- Risk of bias: Allocation concealment (method)
- Risk of bias: Blinding - outcomes assessment
- Risk of bias: Incomplete outcome data (GWG)

We will ignore high risk of bias in "Blinding – staff participants" due to the nature of the interventions and high risk of bias in "Selective reporting" because any data needed for this analysis has been requested from authors regardless of whether it was reported in their publications or not.

These sensitivity analyses will be performed on the primary models without intervention effect modifiers.

7.3.3 Adherence

We will consider excluding participants considered as not adherent if sufficient data is available. These sensitivity analyses will be performed on the primary models without intervention effect modifiers.

7.3.4 Components of TIDiER framework

We will conduct sensitivity analysis based on each of the TIDieR components:

- Theory (Yes, No)
- Intervention Type (Diet, Exercise, and Mixed)
- Resources (Combination, None, Self-monitoring tool, Written resource/other)
- Intervention Facilitator (Allied Health, Fitness Specialist, Medical, Researcher, Unsupervised/ eHealth)
- Prior Training (Yes, No/NR)
- Structure (Group, Individual, Individual & Group);

- Intervention Method (Face to face, Face to Face & Remote, Remote)
- Location (Exercise Centre, Hospital/Antenatal Clinic, Non-Clinical setting)
- Number of sessions (1-5, 6-10, 11-25, >25)
- Duration (0 - 12 weeks, 13 - 24 weeks, 25-40 weeks, NR)
- Weeks of gestation (First trimester, Early second trimester, Late second Trimester, Third trimester)
- Tailoring (Yes, No)

8 ANALYSIS OF SECONDARY OBJECTIVES

8.1 Description of secondary objectives

Secondary objectives are as follows:

1. To evaluate the effects of the interventions on critically important (1) maternal complications such as hypertensive diseases, caesarean section or preterm birth; and (2) offspring complications such as stillbirth, large for gestational age or admission to the neonatal unit in women with gestational diabetes.
2. To categorise the interventions by core components and to undertake network meta-analysis to rank them by effectiveness.
3. To assess the effects of interventions for specific other definitions of gestational diabetes (WHO, International Association of Diabetes and Pregnancy Study Group (IADPSG), modified IADPSG, and American Diabetes Association (ADA)), and on fasting and 2-hour post-prandial glucose levels.
4. To determine the cost-effectiveness of interventions using decision–analytical modelling.

8.2 Secondary analysis

1. We will consider whether lifestyle interventions reduce maternal and offspring outcomes in women with GDM irrespective of whether they have an effect on GDM. We will consider that baseline variables (described in section 5.9) modify this association. For each outcome separately, we will fit a suitable two-stage logistic regression meta-analysis model that accounts for clustering of participants within studies.
2. Network Meta-analysis
3. We will follow the same analysis as Primary objective 1, except that the definition of GDM will change from NICE criteria to include the other definitions (described in section 2.2.2).

9 AMENDMENTS TO VERSION 1.0

N/A

10 Bibliography

[1] D. Coomar *et al.*, “Diet and physical activity in pregnancy to prevent gestational diabetes: A protocol for an individual participant data (IPD) meta-analysis on the differential effects of interventions with economic evaluation,” *BMJ Open*, vol. 11, no. 6, Jun. 2021, doi: 10.1136/bmjopen-2020-048119.

[2] L. A. Stewart *et al.*, “Preferred reporting items for a systematic review and meta-analysis of individual participant data: The PRISMA-IPD statement,” *JAMA - Journal of the American Medical Association*. 2015, doi: 10.1001/jama.2015.3656.

[3] R. D. Riley, J. F. Tierney, and L. A. Stewart, “Individual participant data meta-analysis : a handbook for healthcare research,” p. 550, 2021.

[4] National Institute for Health and Care Excellence, “Diabetes in pregnancy : management from preconception to the postnatal period,” *NICe*, 2015, doi: 978-1-4731-0993-3.

[5] WHO, “World Health Organization: Definition and Diagnosis of Diabetes Mellitus and Intermediate Hyperglycemia. Geneva, World Health Org.,” *WHO2*, 2006, doi: ISBN 92 4 159493 4.

[6] INTERNATIONAL ASSOCIATION OF DIABETES AND PREGNANCY Study Groups.E., “Recommendations on the diagnosis and classification of hyperglycemia in pregnancy.,” in *Diabetes Care*, 2010, doi: 10.2337/dc09-1848.

[7] L. Poston *et al.*, “Effect of a behavioural intervention in obese pregnant women (the UPBEAT study): A multicentre, randomised controlled trial,” *Lancet Diabetes Endocrinol.*, 2015, doi: 10.1016/S2213-8587(15)00227-2.

[8] B. H. Al Wattar *et al.*, “Effect of simple, targeted diet in pregnant women with metabolic risk factors on maternal and fetal outcomes (ESTEEM): Study protocol for a pragmatic multicentre randomised trial,” *BMJ Open*, 2016, doi: 10.1136/bmjopen-2016-013495.

[9] A. Goyal, Y. Gupta, R. Singla, S. Kalra, and N. Tandon, “American Diabetes Association ‘Standards of Medical Care—2020 for Gestational Diabetes Mellitus’: A Critical Appraisal,” *Diabetes Therapy*, vol. 11, no. 8. Adis, pp. 1639–1644, Aug. 2020, doi: 10.1007/s13300-020-00865-3.

[10] “Effect of diet and physical activity based interventions in pregnancy on gestational weight gain and pregnancy outcomes: meta-analysis of individual participant data from randomised trials,” *BMJ*, vol. 358, p. j3119, 2017, doi: 10.1136/bmj.j3119.

[11] E. Rogozińska *et al.*, “Effects of antenatal diet and physical activity on maternal and fetal outcomes: Individual patient data meta-analysis and health economic evaluation,” *Health Technol. Assess. (Rockv).*, vol. 21, no. 41, Aug. 2017, doi: 10.3310/hta21410.

[12] T. C. Hoffmann *et al.*, “Better reporting of interventions: Template for intervention description and replication (TIDieR) checklist and guide,” *BMJ*, vol. 348, Mar. 2014, doi: 10.1136/bmj.g1687.

[13] S. Michie, M. M. van Stralen, and R. West, “The behaviour change wheel: A new method for characterising and designing behaviour change interventions,” *Implement. Sci.*, vol. 6, no. 1, p. 42, Apr. 2011, doi: 10.1186/1748-5908-6-42.

[14] S. T. Brookes, E. Whitley, T. J. Peters, P. A. Mulheran, M. Egger, and G. Davey Smith, “Subgroup analyses in randomised controlled trials: Quantifying the risks of false-positives and false-negatives,” *Health Technology Assessment*, vol. 5, no. 33. National Co-ordinating Centre for HTA, Oct. 2001, doi: 10.3310/hta5330.

[15] E. Kontopantelis, D. A. Springate, R. Parisi, and D. Reeves, “Simulation-based power calculations for mixed effects modeling: Ipdpower in stata,” *J. Stat. Softw.*, vol. 74, no. 1, pp. 1–25, Oct. 2016, doi: 10.18637/jss.v074.i12.

[16] J. Ensor, D. L. Burke, K. I. E. Snell, K. Hemming, and R. D. Riley, “Simulation-based power calculations for planning a two-stage individual participant data meta-analysis,” *BMC Med. Res. Methodol.*, vol. 18, no. 1, p. 41, May 2018, doi: 10.1186/s12874-018-0492-z.

[17] i-WIP Collaborative Group: Centro Rosarino de Estudios Perinatales, “i-WIP. Repository of individual participant data from randomised controlled trials with diet and physical activity based interventions in pregnancy,” 2013. .

[18] T. R. Sullivan, I. R. White, A. B. Salter, P. Ryan, and K. J. Lee, “Should multiple imputation be the method of choice for handling missing data in randomized trials?,” *Stat. Methods Med. Res.*, vol. 27, no. 9, pp. 2610–2626, Sep. 2018, doi: 10.1177/0962280216683570.

[19] I. Ahmed, A. J. Sutton, and R. D. Riley, “Assessment of publication bias, selection bias, and unavailable data in meta-analyses using individual participant data: A database survey,” *BMJ*, 2012, doi: 10.1136/bmj.d7762.

[20] A. E. Ruifrok *et al.*, “Study protocol: Differential effects of diet and physical activity based

interventions in pregnancy on maternal and fetal outcomes-individual patient data (IPD) meta-analysis and health economic evaluation," *Syst. Rev.*, 2014, doi: 10.1186/2046-4053-3-131.
[21] R. D. Riley *et al.*, "One-stage individual participant data meta-analysis models for continuous and binary outcomes: Comparison of treatment coding options and estimation methods," *Stat. Med.*, vol. 39, no. 19, pp. 2536–2555, 2020, doi: 10.1002/sim.8555.
[22] J. E. Cornell *et al.*, "Random-effects meta-analysis of inconsistent effects: A time for change," *Ann. Intern. Med.*, 2014, doi: 10.7326/m13-2886.
[23] D. L. Burke, J. Ensor, and R. D. Riley, "Meta-analysis using individual participant data: one-stage and two-stage approaches, and why they may differ," *Stat. Med.*, 2017, doi: 10.1002/sim.7141.
[24] P. Royston and W. Sauerbrei, "A new approach to modelling interactions between treatment and continuous covariates in clinical trials by using fractional polynomials," *Stat. Med.*, vol. 23, no. 16, pp. 2509–2525, Aug. 2004, doi: 10.1002/SIM.1815.
[25] X. Sun, M. Briel, S. D. Walter, and G. H. Guyatt, "Is a subgroup effect believable? Updating criteria to evaluate the credibility of subgroup analyses," *BMJ*, vol. 340, no. 7751, pp. 850–854, Apr. 2010, doi: 10.1136/BMJ.C117.
[26] J. A. C. Sterne *et al.*, "Recommendations for examining and interpreting funnel plot asymmetry in meta-analyses of randomised controlled trials," *BMJ*, vol. 343, no. 7818, Aug. 2011, doi: 10.1136/BMJ.D4002.
[27] R. D. Riley *et al.*, "Meta-analysis of continuous outcomes combining individual patient data and aggregate data," *Stat. Med.*, 2008, doi: 10.1002/sim.3165.
[28] R. D. Riley and E. W. Steyerberg, "Meta-analysis of a binary outcome using individual participant data and aggregate data," *Res. Synth. Methods*, 2010, doi: 10.1002/jrsm.4.
[29] *Cochrane Handbook for Systematic Reviews of Interventions*. 2019.

11 APPENDIX A – list of IPD studies

Table 3: Trials who have given support to this IPD meta-analysis

Study	Country	Sample size	Intervention
IPD from old studies from BMJ 2017 publication (n=32; 12933 women)			
Althuizen 2012	Netherlands	269	Mixed approach
Barakat 2008	Spain	160	Physical Activity
Barakat 2011	Spain	80	Physical Activity
Barakat 2012a	Spain	320	Physical Activity
Bogaerts 2012	Belgium	205	Mixed approach
Dodd 2014	Australia	2212	Mixed approach
El Beltagy 2013	Egypt	100	Mixed approach
Guelinckx 2010	Belgium	195	Mixed approach
Harrison 2013	Australia	228	Mixed approach
Hui 2011	Canada	224	Mixed approach
Jeffries 2009	Australia	286	Mixed approach
Khoury 2005	Norway	290	Diet
Luoto 2011	Finland	442	Mixed approach
Nascimento 2011	Brazil	82	Physical Activity
Ong 2009	Australia	12	Physical Activity
Oostdam 2012	Netherlands	124	Physical Activity
Perales 2014	Spain	184	Physical Activity
Petrella 2013	Italy	63	Mixed approach
Phelan 2011	USA	401	Mixed approach
Poston 2013	UK	183	Mixed approach

Poston 2015	UK	1555	Mixed approach
Rauh 2013	Germany	250	Mixed approach
Renault 2013	Denmark	425	Mixed approach
Rono 2018	Finland	228	Mixed approach
Rono 2018a	Finland	492	Mixed approach
Ruiz 2013	Spain	962	Physical Activity
Sagedal 2016	Norway	606	Mixed approach
Stafne 2012	Norway	855	Physical Activity
Vinter 2011	Denmark	360	Mixed approach
Vitolo 2011	Brazil	315	Diet
Walsh 2012	Ireland	759	Diet
Wolff 2008	Denmark	66	Diet
**IPD from new studies (n=24; 17988 women)**			
Al Wattar 2019	UK	3225	Diet
Chao 2017	USA	41	Mixed approach
Arthur 2020	Australia	400	Mixed approach
Assaf-Balut 2017	Spain	1000	Diet
Barakat 2016	Spain	800	Physical Activity
Barakat 2018	Spain	508	Physical Activity
Bisson 2015	Canada	50	Physical Activity
Bruno 2016	Italy	191	Diet
Cordero 2014	Spain	257	Physical Activity
Dekker 2015	Australia	43	Physical Activity
Dodd 2019	Australia	633	Mixed approach
Garmendia 2020	Chile	4631	Mixed approach
Garnaes 2016	Norway	91	Physical Activity
Hawkins 2014	USA	68	Mixed approach
Hui 2014	Canada	116	Mixed approach
Kennelly 2018	Ireland	565	Mixed approach
Kunath 2019	Germany	2261	Mixed approach
McCarthy 2016	Australia	382	Diet
Menchini 2020	Italy	82	Diet
Nobles 2018	USA	290	Physical Activity
Olson 2018	USA	1689	Mixed approach
Peleaz, 2019	Spain	301	Physical Activity
Phelan 2018	USA	264	Mixed approach
Willcox 2017	Australia	100	Mixed approach
**IPD from all studies (n=56; 30921 women)**			

12 Appendix B – template results tables for main report

(1) Descriptive

Study-level characteristics

Study information

	Year of publication	Country	Sample size	Randomization	Overall risk of bias*
Althuizen					
...					
…					

* High risk of bias is defined as either at least one high risk in items 1 (randomisation), 2 (Allocation concealment), 4 (Blinding outcomes assessment) and 5 (Incomplete outcome data) or not a single item of low risk.

Participant-level characteristics

Participant-level characteristics for each study can be found in the report appendix

Study	Baseline characteristics	Intervention	Control
**All Diet**	N studies	4	
	N obs	400	500
	Age, Mean(SD)		
	Height		
	...		
	EQ5D		
**All Exercise***	**…**		
**All Mixed***	**…**		
**All**	**…**		

The Renault study has 2 intervention arms and therefore appears once in the exercise and once in the mixed intervention group.

(2) Primary analysis

Analysis results for each study can be found in the appendix
Effect modifiers are defined as follows:

- BMI, continuous scale
- Age in years, continuous scale
- Ethnicity, binary: caucasion vs non-caucasion
- Parity, binary: nulliparous vs not nulliparous
- Socioeconomic status, categorical (proxy variable 'Education'): low vs middle vs high
-

We will present the tables given below for each of the different interventions (all, diet only, exercise only and mixed)

Two-stage meta-analysis of GDM: logistic regression

	Sample size (Number of studies)	Intervention group N (%)	Control Group N (%)	Adjusted odds ratio*, 95% CI	Treatment-covariate interaction
Primary analysis 1a: Two-stage meta-analysis of GDM					
Pooled Effect	9000 (33)	6.1 (1.2)	9.5 (1.1)	3.3 (1.3, 5.3)	n/a
Primary analysis 1b: Two-stage meta-analysis of GDM, effect modifier Baseline BMI					
Pooled Effect					
Primary analysis 1c: Two-stage meta-analysis of GDM, effect modifier Age					
Pooled Effect					
Primary analysis 1d: Two-stage meta-analysis of GDM, effect modifier Ethnicity					
Pooled Effect					
Primary analysis 1e: Two-stage meta-analysis of GDM, effect modifier Parity					
Pooled Effect					
Primary analysis 1e: Two-stage meta-analysis of GDM, effect modifier Socioeconomic status					

* Model adjusted for age, baseline BMI and clustering effect

One–stage meta-analyses

	Univariate analyses		Multivariate analysis N=x (x studies)
	**Sample size (Number of studies)**	**Adjusted odds ratio*, 95% CI**	**Adjusted odds ratio*, 95% CI**
Pooled Effect Age	20000 (60)		
Pooled Effect Caucasian			
Pooled Effect Baseline DM			
Pooled Effect Parity			
Pooled Effect Education:<br>• Low (reference category)<br>• Medium<br>• High	8000 (30)<br>1000 (25%)<br>2000 (50%)<br>1000 (25%)		
Pooled Effect Baseline Activity			
Pooled Effect Mental Health			

* Model adjusted for age, baseline BMI and clustering effect

(3) Sensitivity analyses to primary analyses

Sources of bias: small study effects and publication bias
Colour enhanced funnel plots
Eggers test
Peters test

Other sources of bias investigated:
1) Unavailable IPD
2) Study quality
3) Duration of intervention
4) Adherence, if applicable
5) Components of TIDieR framework

	Sample size (Number of studies)	Intervention group N (%)	Control Group N (%)	Adjusted odds ratio*, 95% CI
1) Two-stage meta-analysis of GDM including aggregate data				
Pooled Effect	20000 (60)			
2) Two-stage meta-analysis of GDM excluding studies with high risk of bias				
Pooled Effect	5000 (18)			
3) Two-stage meta-analysis of GDM excluding studies with duration of intervention <xx weeks				
Pooled Effect				
4) Two-stage meta-analysis of GDM excluding studies with adherence < xx				
Pooled Effect				
5) Two-stage meta-analysis of GDM by components of TIDieR framework				

* Model adjusted for age, baseline BMI and clustering effect

(4) Secondary analyses

13 Appendix C – Data requirements for health economic modelling

Appendix 4: Characteristics of included studies for the individual participant data (IPD) meta-analysis of lifestyle interventions on gestational diabetes

a) Studies contributing IPD (n=54)

Study	Country	Continent	Intervention	Intervention key components	Comparison	Baseline BMI Category	Number of Women
Al Wattar 2019	UK	Europe	Diet	Mediterranean diet + mixed nuts and extra virgin olive oil with tailored dietary advice	Dietary advice as per UK national recommendations for antenatal care and weight management in pregnancy	All BMI groups	1252
Arthur 2020	Australia	Oceania	Mixed	Tailored IOM guideline weight gain coaching + weight diary + weighing scales	Standard IOM written and verbal information regarding appropriate weight gain in pregnancy.	All BMI groups	396
Assaf-Balut 2017	Spain	Europe	Diet	Mediterranean diet recommendation + EVOO + pistachio nuts as advised by Dietician	Controls were advised to restrict consumption of dietary fat, including EVOO and nuts as advised by Midwife	All BMI groups	1000
Baciuk 2008	Brazil	South America	Physical Activity	Moderate water aerobics	No intervention – usual care	All BMI groups	70

**Study**	**Country**	**Continent**	**Intervention**	**Intervention key components**	**Comparison**	**Baseline BMI Category**	**Number of Women**
Barakat 2008	Spain	Europe	Physical Activity	Toning and very mild resistance exercise	Women asked to maintain their usual level of activity	All BMI groups	160
Barakat 2011	Spain	Europe	Physical Activity	Toning and very mild resistance exercise	Standard Care	All BMI groups	67
Barakat 2012a	Spain	Europe	Physical Activity	Exercises for arms and abdomen, and aerobic dance to improve posture, strengthen muscles of labour and pelvic floor and prevent lower back pain	Standard Care	All BMI groups	279
Barakat 2016	Spain	Europe	Physical Activity	Aerobic exercise, aerobic dance, muscular strength, and flexibility, to meet ACOG standards	General advice from healthcare provider about benefits of physical activity	All BMI groups	840
Barakat 2018	Spain	Europe	Physical Activity	Aerobic exercises, dance, strength training, and flexibility to meet ACOG standards	Standard care – general nutritional and physical activity advice – asked about exercise habits once every trimester	All BMI groups	429
Bisson 2015	Canada	North America	Physical Activity	Progressive cardiovascular training, muscular exercises	Given general advice on benefits	BMI $\geq$ 30	45

Study	Country	Continent	Intervention	Intervention key components	Comparison	Baseline BMI Category	Number of Women
				for upper and lower limbs, and a cool-down	of physical activity and were not discouraged from doing physical activity although no specific physical activity intervention		
Bogaerts 2012	Belgium	Europe	Mixed	Antenatal lifestyle intervention sessions which focused on energy balance, energy expenditure, and physical activity	Routine antenatal care as per national guideline ‘prenatal care’	BMI ≥ 30	197
Bruno 2016	Italy	Europe	Mixed	A personalized diet with a daily intake of 1500 kcal/day. The physical intervention encouraged pregnant women to engage in moderate-intensity activity for at least 30 minutes, three times a week	Standard physical activity recommendations. Women were asked about their adherence to the suggested lifestyle	BMI ≥ 25	131
Chao 2017	USA	North America	Mixed	Telephone counselling sessions focused on weight management through nutrition, exercise, and lifestyle changes, aimed at a calorie increase of only 300	Standard counselling on nutrition, exercise and weight gain goals at obstetric visits. No telephone and WiFi weighing	BMI ≥ 25	41

Study	Country	Continent	Intervention	Intervention key components	Comparison	Baseline BMI Category	Number of Women
				per day above pre-pregnancy levels	intervention. All participants completed monthly 24-hour food recalls using online system		
Cordero 2014	Spain	Europe	Physical Activity	Exercises focused on low-impact aerobics, muscle strengthening (avoiding abdominal focus), pelvic floor training, and flexibility, with intensity monitored to ensure maternal safety. Water-based sessions included swimming, strength exercises, and relaxation, all performed under professional supervision.	Women in control group remained inactive	All BMI groups	247
Dekker 2015	Australia	Oceania	Physical Activity	Group education session with individualized exercise plans. Monthly physiotherapist consultations and self-monitoring diaries	Control participants attended standard group education session, receiving information on exercise, nutrition and adequate GWG.	BMI ≥ 30	35

Study	Country	Continent	Intervention	Intervention key components	Comparison	Baseline BMI Category	Number of Women
Dodd 2014	Australia	Oceania	Mixed	Dietary and lifestyle intervention led by a research dietician and trained assistants, focusing on balanced nutrition and increased physical activity.	Standard care following local hospital guidelines without routine advice on diet, exercise or gestational weight	BMI ≥ 25	2199
Dodd 2019	Australia	Oceania	Mixed	Tailored dietary and activity plans aligned with Australian guidelines, SMART goal setting, and ongoing support. Women received written materials, recipes, and menu plans to promote balanced nutrition, reduced energy-dense foods, increased fiber, and realistic, measurable lifestyle changes.	Received standard antenatal care according to hospital guidelines without information being given on diet, physical activity or weight gain information.	BMI 18.5 - 24.9	641
El Beltagy 2013	Egypt	Africa	Mixed	Mild physical activity programme and diet modification	No details	BMI ≥ 30	93
Garmendia 2020	Chile	South America	Mixed	Counselling pregnant women on diet, physical activity, and GWG targets, and offering supervised exercise classes. Dietitian referrals were provided for excessive weight	Provided with standard routine antenatal care and nutritional counselling in accordance with	BMI 18.5 - 24.9	4631

Study	Country	Continent	Intervention	Intervention key components	Comparison	Baseline BMI Category	Number of Women
				gain based on IOM 2009 guidelines, with an emphasis on healthy nutrition, breastfeeding benefits, and moderate-intensity physical activity.	national guidelines but no further advice on adequate GWG was given.		
Garnaes 2016	Norway	Europe	Physical Activity	Treadmill walking/jogging, resistance training, and pelvic floor exercises, alongside a weekly home exercise program and daily pelvic floor exercise	Standard maternity care following Norwegian guidelines given by healthcare providers. They were encouraged to continue their regular activities, including exercise.	BMI ≥ 25	91
Guelinckx 2010	Belgium	Europe	Mixed	A brochure on diet, physical activity, and GWG management, one-hour counselling sessions by a nutritionist who guided on a balanced diet, energy balance, and behavioural modifications to limit energy-dense foods and increase healthier alternatives, with physical activity	No intervention	BMI ≥ 30	195

Study	Country	Continent	Intervention	Intervention key components	Comparison	Baseline BMI Category	Number of Women
Harrison 2013	Australia	Oceania	Mixed	Healthy eating, physical activity, and behaviour change strategies based on social cognitive theory	A single brief education session based on Australian Dietary and Physical Activity Guidelines was provided along with written versions of guidelines. GWG was not discussed	BMI ≥ 25	238
Hawkins 2014	USA	North America	Mixed	Counselling sessions on moderate-intensity activity and reducing saturated fats and increasing fibre through personalised goals, tracking tools, and culturally sensitive materials	Standard Care	BMI ≥ 25	68
Hui 2011	Canada	North America	Mixed	Community-based exercise program with group sessions for walking, aerobic, stretching, and strength exercises + dietary counselling sessions from registered dietitians, focusing on personalised advice based on food choice, weight gain	Standard care given according to Society of Obstetricians and Gynaecologists of Canada. Exercise and dietary intervention not provided.	All BMI groups	183

Study	Country	Continent	Intervention	Intervention key components	Comparison	Baseline BMI Category	Number of Women
Hui 2014	Canada	North America	Mixed	Exercise program with aerobic, stretching, and strength exercises, delivered in group sessions or via DVD for home use, along with a dietary intervention involving two personalised consultations	Received standard prenatal care and a package of information on healthy habits during pregnancy from Health Canada.	All BMI groups	113
Jeffries 2009	Australia	Oceania	Mixed	Personalised weight measurement card + counselling	No intervention	All BMI groups	282
Kennelly 2018	Ireland	Europe	Mixed	Educational sessions	Received standard antenatal care - in Ireland this does not include uniform advice on diet, exercise or weight gain during pregnancy	BMI ≥ 25	565
Khaledan 2010	Iran	Asia	Physical Activity	Modified Clapp exercise program which included stretching, aerobic exercise tailored to 60% of the maximum heart rate, hydration breaks, and a	Received dietary information based on Food Pyramid guidelines recommended by American Agricultural	All BMI groups	39

Study	Country	Continent	Intervention	Intervention key components	Comparison	Baseline BMI Category	Number of Women
				cooling-down phase with light stretching.	Department. Iron and Folic acid tablets provided as part of routine pregnancy care		
Khoury 2005	Norway	Europe	Diet	A cholesterol-lowering diet	Control group advised to consume usual diet.	All BMI groups	289
Kunath 2019	Germany	Europe	Mixed	Counselling sessions by trained healthcare professionals, focusing on balanced diet, physical activity, and self-monitoring of weight gain using IOM-based weight-gain charts	Received information leaflets on healthy pregnancy lifestyle and routine prenatal care.	BMI 18.5 - 40.0	2261
Luoto 2011	Finland	Europe	Mixed	Counselling sessions and monthly thematic group meetings, promoting adherence to IOM weight-gain guidelines, Finnish dietary recommendations, and 800 MET minutes of weekly physical activity. Personalized action plans, BMI-specific weight-gain charts, and dietary and physical activity	Routine care including usual dietary and physical activity counselling	BMI ≥ 25	395

Study	Country	Continent	Intervention	Intervention key components	Comparison	Baseline BMI Category	Number of Women
McCarthy 2016	Australia	Oceania	Diet	Session providing dietary advice, serial self-weighing guidance, and IOM-based weight-gain targets	Standard care group given a card listing their booking BMI and recommended gestational weight gain. No other changes made to routine care.	BMI ≥ 25	382
Nascimento 2011	Brazil	South America	Physical Activity	Sessions guided by a physical therapist, following a standard protocol of stretching, limb-strengthening, and relaxation exercises, along with home exercise counselling to perform protocol exercises or walking five times weekly,	Routine antenatal advice and standard nutritional counselling. Not provided with physical activity counselling.	BMI ≥ 25	82
Olson 2018	USA	North America	Mixed	Website offering behaviour change tools, including a weight gain tracker, goal-setting and self-monitoring tools for diet and physical activity, health information, and reminders.	Placebo control group also had access to a control website but not a weight gain tracker and diet/physical activity tools.	BMI 18.5 – 34.9	1126
Ong 2008	Australia	Oceania	Physical Activity	Home-based stationary cycling program	No intervention	All BMI groups	13

**Study**	**Country**	**Continent**	**Intervention**	**Intervention key components**	**Comparison**	**Baseline BMI Category**	**Number of Women**
Oostdam 2012	The Netherlands	Europe	Physical Activity	Exercise programme of aerobic and strength training, provided using cycle ergometers, treadmills, cross-trainers and rowing machines.	Usual care by midwives and obstetricians	BMI ≥ 25	105
Pelaez 2019	Spain	Europe	Physical Activity	Group-based exercise sessions including an 8-minute warm-up, 35 minutes of low-impact aerobics and resistance training targeting major muscle groups, and a 15-minute cooldown with pelvic floor exercises, stretching, and relaxation	Basic care provided by midwives and obstetricians. General nutrition and physical activity counselling, not being discouraged from exercising on their own accord.	All BMI groups	345
Perales 2014	Spain	Europe	Physical Activity	Group exercise sessions comprising warm-up, aerobic dance, resistance, balance, pelvic floor muscle training, and cooldown exercises.	Standard care	All BMI groups	165
Petrella 2013	Italy	Europe	Mixed	The calorie allowance was 1500 kcal/day with an extra 200 kcal/day for obese women and 300 kcal/day for overweight women to account for physical activity programme. The target diet	Control group received a nutritional booklet based on Italian guidelines for a	BMI ≥ 25	61

Study	Country	Continent	Intervention	Intervention key components	Comparison	Baseline BMI Category	Number of Women
				composition was 55% carbohydrate, 20% protein, and 25% fat given as three main meals and three snacks. The last snack was 2 hours after dinner to prevent overnight hypoglycaemia. The minimum recommended intake of carbohydrates was 225 g/day. The exercise intervention was 30 minutes of moderate intensity activity for a minimum of 3 days a week.	healthy diet during pregnancy		
Phelan 2011	USA	North America	Mixed	Sessions on appropriate GWG targets, physical activity (30 minutes of walking), and calorie intake (20 kcal/kg). Daily self-monitoring of weight, limiting high-fat foods. Weight scales, food diaries and pedometers provided	Standard scheduled visits providing nutritional counselling. Study newsletters related to pregnancy given at 2-monthly intervals. Women weighed regularly but not given weight graphs	All BMI groups	393
Phelan 2018	USA	North America	Mixed	Gradually increase pregnant women's daily steps until they reached a goal of 10,000 steps	received the typical prenatal care offered by their	BMI ≥ 25	264

Study	Country	Continent	Intervention	Intervention key components	Comparison	Baseline BMI Category	Number of Women
				per day + Daily tracking of food and drink intake, calorie counting, and physical activity	providers, including physicians, nurses, nutritionists, and counsellors from the Women, Infants, and Children’s Special Supplemental Nutrition Program (WIC).		
Poston 2013	UK	Europe	Mixed	Exchanging high GI foods for low GI foods + increase and maintain daily physical activity level	Control group attended data collection appointments with study midwives, coinciding with routine antenatal visits	BMI ≥ 30	164
Poston 2015	UK	Europe	Mixed	Increase pedometer steps and daily activity incrementally; moderate activity in the form of walking in line with UK RCOG recommendations, + healthier eating with no restriction of calories, substitute low glycaemic index for medium/high glycaemic index food, restrict	Routine antenatal care, explaining the risks of obesity, advising on a healthy diet and safe levels of physical activity.	BMI ≥ 30	1554

Study	Country	Continent	Intervention	Intervention key components	Comparison	Baseline BMI Category	Number of Women
				sugar-sweetened beverages but not fruits and reduce saturated fatty acid intake.			
Rauh 2013	Germany	Europe	Mixed	General lifestyle advice including nutrition, physical activity and appropriate GWG	Routine antenatal care including an information leaflet consisting of 10 general statements on a healthy lifestyle during pregnancy not including advice on diet or gaining weight	All BMI groups	244
Renault 2013	Denmark	Europe	Mixed	A low-fat low-calorie (1200–1675 kcal/day) Mediterranean-style diet, with preference to fish and oils as per Danish national guidelines for healthy eating + increase physical activity aiming for a daily step count of 11,000/day	Standard care including one consultation with a dietician. Dietary advice given as per Danish guidelines for healthy eating. Only oral advice given and women asked to aim for a GWG of <5kg	BMI ≥ 30	425
Rono 2018	Finland	Europe	Mixed	A minimum of 150 minutes physical activity per week at	Completed same number of visits to	BMI ≥ 30	128

Study	Country	Continent	Intervention	Intervention key components	Comparison	Baseline BMI Category	Number of Women
				moderate-intensity and adopt an overall active lifestyle + consume healthy foods and reduce the intake of sugar-rich foods.	study nurse, questionnaires and measurements as intervention group. Given information leaflets on healthy diet and physical activity, receiving usual Finnish antenatal care.		
Ruiz 2013	Spain	Europe	Physical Activity	Supervised physical activity sessions of light to moderate intensity	Standard usual care with visits to midwives and obstetricians. Information provided on nutrition and physical activity and women were not discouraged from exercising	All BMI groups	927
Sagedal 2016	Norway	Europe	Mixed	Diet recommendations focus on 10 key recommendations based on Norwegian directorate of health guidance. Exercise sessions consisting of strength training and moderate cardiovascular exercises, and	Standard prenatal care	All BMI groups	600

Study	Country	Continent	Intervention	Intervention key components	Comparison	Baseline BMI Category	Number of Women
				20 minutes warm-up and stretching			
Stafne 2012	Norway	Europe	Physical Activity	Exercise programme including aerobic activity, strength training and balance exercises supervised by a physiotherapist	Usual care – not discouraged from exercising. Provided with written recommendations on diet, pelvic floor exercises and pregnancy related lumbopelvic pain	All BMI groups	854
Vinter 2011	Denmark	Europe	Mixed	Dietary counselling to limit GWG in pregnancy to 5 kg and moderate physical activity lasting 30–60 minutes	Access to a website with advice on diet and physical activity during pregnancy	BMI ≥ 30	304
Vitolo 2011	Brazil	South America	Diet	Dietary counselling + restriction on consumption of foods rich in fat and cooking oils	Did not received dietary guidelines but were informed about nutritional status and asked to carry on with their usual prenatal care	All BMI groups	301
Walsh 2012	Ireland	Europe	Diet	One 2-hour dietary education session with a dietitian + focus	Routine antenatal care with no	All BMI groups	759

Study	Country	Continent	Intervention	Intervention key components	Comparison	Baseline BMI Category	Number of Women
				on consumption of low glycaemic index food	specific dietary recommendations or advice about GWG		
Willcox 2017	Australia	Oceania	Mixed	Four to five individually tailored, interactive text messages per week with encouragement of positive health behaviours, monitoring of individual goals and encouragement of self-monitoring of GWG	Usual care – given information brochures including advice on diet and physical activity prior to first visit and were encouraged to be weighed at first visit.	BMI ≥ 25	100
Wolff 2008	Denmark	Europe	Diet	10-hour consultation on Danish dietary recommendations based on individual energy requirement and delivered by dietitian	No Intervention	BMI ≥ 30	59

b) Studies that did not contribute IPD (n=50)

Study	Country	Continent	Intervention	Key component	Comparison	BMI Category	Number of Women
Abdel-Aziz 2018	Egypt	Africa	Diet	Counselling session with nutritionist + advice on healthy weight gain based on dietary guidelines to improve eating habits, educate on healthier food choices	Participants of the control group received standard maternity care. Women received standard nutrition counselling provided by the physicians and nurses based on the Maternal and child Health Program components.	All BMI groups	147
Barakat 2012	Spain	Europe	Physical Activity	Physical conditioning program made up of land and water aerobics	Standard care	All BMI groups	83
Barakat 2013	Spain	Europe	Physical Activity	Aerobic, strength, and flexibility exercises	The control group received general advice on physical activity from their midwife and had the same prenatal care and visits with healthcare providers (midwives, obstetricians and family doctors) as the exercise group. They were not discouraged from exercising on their own.	All BMI groups	428
Barakat 2014	Spain	Europe	Physical Activity	Walk and static stretching, toning and joint mobilization exercises, aerobic dance, and specific exercises	During this period, women in the control group did not engage in physical activity and solely relied on	All BMI groups	200

				targeting major muscle groups in the legs, buttocks, and abdomen to stabilize the lower back	information from their midwives or healthcare providers.		
Barakat 2019	Spain	Europe	Physical Activity	Gradual warm-up, aerobic exercises, muscle strengthening, coordination, stretching, pelvic floor strengthening, relaxation, and a final talk	Pregnant women in the standard care group attended regular visits with obstetricians and midwives, (according to Hospital protocol), usually every 4–5 weeks until the 36–38th week of gestation and then weekly until delivery. They received counselling on nutrition and physical activity and were asked about their exercise habits once each trimester using a "Decision Algorithm" by telephone.	All BMI groups	456
Barquiel 2023	Spain	Europe	Mixed	Educational session about an adequate diet and physical activity given by nurses with expertise in obesity and diabetes	Control group received information about diet quality, physical activity and were informed about the concept of adequate gestational weight gain based on the 2009 Institute of Medicine (IOM) recommendations.	BMI ≥ 30	169
Brownfoot 2016	Australia	Oceania	Mixed	Weighing at each antenatal clinic appointment + counselling according to the IOM gestational weight gain guidelines	Standard antenatal care - recording weight at booking and then at 36 weeks	All BMI groups	741

Buckingham 2019	USA	North America	Mixed	Behavioural lifestyle intervention that included counselling and a wearable fitness tracker including dietary software to increase physical activity and modify carbohydrate intake	Participants in the usual care group attended routine prenatal visits with their healthcare providers. No additional lifestyle counselling was provided to this group.	BMI 18.5 - 24.9	47
Cahill 2018	USA	North America	Mixed	Cognitive-behavioural lifestyle program. This included goal setting for appropriate GWG, regular self-assessment, education, observational learning through role-play, and home environment changes.	Participants assigned to the standard care curriculum had home visits focused on development-centered parenting support and education and parent-child interaction using a family strength-based approach.	BMI ≥ 25	240
Chan 2018	Hong Kong	Asia	Mixed	Dietitian-led lifestyle and trainer led exercise program with individualised menu plan and healthy lifestyle booklets to achieve a varied, balanced diet emphasising fruit and vegetable consumption, moderate-carbohydrate, low-fat, low-glycaemic index (GI), and low-caloric products in	The control group received routine antenatal care. During each antenatal visit, nurses monitored the body weight of pregnant women. They were also given an educational booklet with dietary and exercise recommendations for pregnancy. Optional antenatal classes were offered, subject to availability.	BMI ≥ 25	220

				appropriate portions + low-impact aerobic exercise			
Da Silva 2017	Brazil	South America	Physical Activity	Moderate-intensity exercise sessions consisting of aerobic activities, strength training and stretching exercises according to the ACOG recommendations	Women in the control group received standard antenatal care and were encouraged to continue their normal daily activities.	BMI < 35	639
Deng 2022	China	Asia	Mixed	Personalized diet and exercise program which included educational manuals and recording of a diet and exercise diary.	The control group only received routine health management until the birth of the baby.	BMI ≥ 25	84
Ding 2021	China	Asia	Mixed	Monthly personalized nutrition care + at least 6000 steps a day	There were no requirements for the control group.	BMI ≥ 25	215
Eslami 2018	Iran	Asia	Mixed	Session on nutrition and physical activity in overweight and obese females	The control group did not receive any educational booklet (standard care)	BMI ≥ 25	140
Ferrara 2020	USA	North America	Mixed	Lifestyle intervention program adapted from the Diabetes Prevention Program, delivered through telehealth. The	Women in the control group received standard Kaiser Permanente Northern California (KPNC) antenatal medical care, which included an antenatal visit at 7–10 weeks' gestation, an additional	BMI ≥ 25	394

				goal of the program was for women to gain weight within the Institute of Medicine guidelines range	seven antenatal visits on average, and periodic health education newsletters, including the Institute of Medicine GWG guidelines and information on healthy eating and physical activity in pregnancy.		
Flanagan 2025	USA	North America	Mixed	A fully remote lifestyle intervention program that included utilisation of meal plans, shopping lists and recipes as well as exercise videos to complete 150 mins of physical activity per week and goals for daily number of steps tracked by a Fitbit	The Usual Care Group received all aspects of the Louisiana Women, Infants and Children program, a brief monthly call to bond them with the trial and access to a private Facebook group where interventionists shared weekly posts unrelated to weight.	BMI 18.5 – 40	351
Gonzalez-Plaza 2022	Spain	Europe	Physical Activity	10,000 steps a day equivalent to 30 minutes per day of moderate physical activity as recommended by the American College of Obstetricians and Gynaecologists	Pregnant women in the control group received oral and written support material. They were advised to perform moderate physical activity for 30 minutes a day, at least 5 days a week, and aim for a GWG between 5-9 kg, according to the IOM. Midwives provided instructions to achieve the physical activity goal gradually and recommended a balanced (Mediterranean) diet of 1800 kcal.	BMI ≥ 30	150

Hajian 2020	Iran	Asia	Mixed	Guidance on healthy eating and exercise with recommendation on diets, weight gain, and physical activity	The control group was evaluated solely for energy intake and level of physical activity and did not receive any further advice or intervention from the researcher.	BMI ≥ 25 - 29.9	66
Hawley 2024	USA	North America	Mixed	Group prenatal care sessions about nutrition, physical activity, mental health, stress, and sexual well-being. Weight tracking was adapted culturally for Samoan women	Routine prenatal care visits, as scheduled by their providers at either the Tafuna Family Health Centre or Lyndon B. Johnson Tropical Medical Centre.	BMI ≥ 26	75
Herring 2016	USA	North America	Mixed	Diet-based personalised daily texts to build self-efficacy and skills, self-motivational texts to achieve daily exercise goals + pedometers and a walking DVD to promote activity	Women receiving usual care at Temple University received standard obstetrical care, and the American College of Obstetricians and Gynecologists information about optimal weight gain during pregnancy.	BMI ≥ 25	56
Horn 2018	USA	North America	Mixed	The DASH diet, modified for pregnant women + encouraged to engage in more than 30 minutes of activity or walk more than 10,000 steps per day and sleep for 7 to 9 hours daily	Usual-care participants received access to the MOMFIT website, which included dietary guidelines and pregnancy recommendations the American College of Obstetrics and Gynecology. They also received biweekly newsletters on pregnancy and infant care.	BMI ≥ 25	281

Jing 2015	China	Asia	Mixed	Education manual on diet and physical activity	The control group received only conventional interventions, such as standard health education manuals produced by the hospital.	All BMI groups	221
Ko 2014	USA	North America	Physical Activity	Moderate to vigorous exercise program.	The women in the control group did not participate in any instructional sessions with the exercise interventionist and did not receive any motivational mailings. They were advised to maintain their usual levels of physical activity, but were not instructed to stop or avoid exercising	All BMI groups	1124
Kong 2014	USA	North America	Physical Activity	Treadmill use to follow the 2008 U.S. physical activity guidelines	Women in the control group were not given physical activity recommendations but were not restricted from participating in PA during pregnancy.	BMI ≥ 25	37
Korpi-Hyovalti 2012	Finland	Europe	Diet	Dietary goals of carbohydrates at 50-55% of energy, fat at 30%, saturated fat less than 10%, and protein at 15-20%, with at least 15g of dietary fibre per 4184 kJ. The recommended	The women were given general information by a nurse on diet and physical activity in a single session to decrease the risk of GDM during pregnancy. The advice was provided both verbally and in writing.	All BMI groups	54

				energy intake was 126 kJ/kg per day for normal-weight women and 105 kJ/kg per day for overweight women.			
Li 2024	China	Asia	Diet	Online diet videos and in-person meal-planning discussions involving weekly weight monitoring, prenatal nutrition and real-time dietary guidance.	Standard antenatal care common for Chinese pregnant women	BMI 18.5 - 24	86
Liu 2022	USA	North America	Mixed	Moderate-intensity PA and follow a diet high in fruits, vegetables, and whole grains while low in saturated and trans fats	The participants of this group were advised to attend their prenatal care provider's clinic visits. To ensure their engagement and retention throughout the program, the group was provided with 6 monthly mailings and 10 weekly podcasts, which were publicly available online. These podcasts mainly focused on healthy pregnancy and fetal development, and were similar in duration and frequency to the ones provided to the intervention group. The mailings and podcasts did not discuss any topics related to weight, physical activity, or diet.	BMI ≥ 25	217
McDonald 2021	USA	North America	Physical Activity	Supervised aerobic exercise on treadmill	Participants in the control group were given the option to participate in low intensity stretching sessions with a	BMI 18.5 - 24.9	74

					focus on major muscle groups. These sessions involved standing or sitting stretches and included breathing techniques. Both exercise and stretching/breathing sessions were supervised and held at one of two university-affiliated gyms.		
Michalopoulou 2023	UK	Europe	Diet	Providing 130–150 g total carbohydrate/day. Refrain from sugary foods and drinks; Reduce portions of starchy carbohydrates; And replace refined starchy carbohydrates with unrefined varieties, and sugary foods/drinks with low/no-sugar alternatives. The consultation was combined with a structured self-help booklet. Participants also received recipes and materials	Routine antenatal care, which, according to national guidance, should include one-off face-to-face routine care NHS healthy eating and food safety advice, during the booking appointment with a midwife, prior to randomization in the study. Participants received no additional advice beyond this.	BMI ≥ 30	51
Okesene-Gafa 2019	New Zealand	Oceania	Diet	Handbook with information on healthy foods, recipes, managing cravings, and staying	Women allocated to routine dietary advice received the New Zealand Ministry of Health pamphlets “Eating for healthy pregnant women” and	BMI ≥ 30	230

				active + home-based education sessions from a community health worker. Also received motivational text messages 3 times a week.	"Healthy weight-gain in pregnancy," with no dietary input from community health workers or text messages.		
Parat 2019	France	Europe	Mixed	Sessions on healthy diets and the benefits of physical activity during pregnancy	The women in the control group received general information about diet and exercise during a face-to-face meeting with a dietician at 26 weeks' gestation. They were provided with the national booklet on nutrition during pregnancy (www.mangerbouger.fr/PNNS/Guides-et-documents/Guides-nutrition).	BMI ≥ 25	206
Perales 2016a	Spain	Europe	Physical Activity	Walking and static stretching of most muscle groups	Standard care	All BMI groups	241
Polley 2002	USA	North America	Mixed	Information on healthy weight gain, exercise during pregnancy, and healthy eating during pregnancy. Women exceeding recommended weight gain received individualized nutrition and behavioural counselling.	Participants in the standard prenatal care group only received the nutrition counselling provided by the doctors, nurses, nutritionists, and Women, Infants and Children counsellors at Magee-Women's Hospital. The focus of this counselling was to promote a well-balanced diet and encourage the use of multivitamin/iron supplements. The research staff did not provide any	BMI ≤ 30	110

					additional information or counselling to this group.		
Price 2012	USA	North America	Physical Activity	Aerobic training program	Control group didn't exercise, only exerted as needed for work/household chores. Told not to exercise to keep group distinction. Tested every 6 weeks after randomization. Dietary advice followed, no calorie estimation.	All BMI groups	62
Quinlivan 2011	Australia	Oceania	Diet	brief dietary interventions by a food technologist + psychological assessments	Women in the control group were allocated to routine public antenatal care. This consisted of midwifery, obstetrician and general practitioner antenatal clinics, with access to high-risk antenatal clinics if indicated on medical grounds.	BMI ≥ 25	124
Rakhshani 2012	India	Asia	Physical Activity	Yoga session consisting of breathing exercises, yogic postures, and meditative exercises	The control group, on the other hand, was offered standard care alongside walking for half an hour in the morning and evening, which was the usual antenatal exercise provided by the hospital during the same period.	All BMI groups	68

Roland 2023	Denmark	Europe	Physical Activity	Gym and swimming pool sessions	Four individual and three group physical activity motivational counselling sessions of 1–2 h duration during pregnancy and a personalized text message once weekly to motivate to increased physical activity.	BMI 18.5 - 24.9	219
Sadiya 2022	UAE	Asia	Mixed	Individualised dietary consultation + optimizing participants' consumption of whole grains, vegetables, fruits, portion control, lowering intake of ultra-processed food, and simple sugars	Standard antenatal care, including general advice regarding lifestyle changes	BMI $\geq$ 30	63
Seneviratne 2016	New Zealand	Oceania	Physical Activity	Magnetic stationary bicycle sessions	The control group was not prescribed an exercise intervention or provided with heart rate monitors.	BMI $\geq$ 25	74
Simmons 2016	United Kingdom, Ireland, Netherlands, Austria, Poland, Italy, Denmark, and Belgium	Europe	Mixed	Aerobic and resistance physical activities	Not specified	BMI $\geq$ 30	436

Teoule 2024	Germany	Europe	Mixed	One-on-one virtual health-coaching sessions and had access to the Buddy Healthcare app. The structured health-coaching sessions included giving advice on a healthy lifestyle such as physical activity and a healthy nutrition but also provided the opportunity to talk about pregnancy symptoms, worries and fears	Standard care,<br>They received a fitness tracker to wear on their wrist (Huawei 6) to self-monitor physical activity and agreed to wear the device during the day for the whole duration of their pregnancy.	All BMI groups	97
Thornton 2009	USA	North America	Mixed	Balanced nutritional regimen + walking target recommendation	Standard Care	BMI ≥ 30	232
Tomic 2013	Croatia	Europe	Physical Activity	Aerobic exercise	The women in the control group were instructed not to engage in any kind of exercise except daily routine activities and received standard antenatal care.	All BMI groups	334
Trak-Fellermeier 2020	Puerto Rico	North America	Mixed	Reducing sedentary behaviours by encouraging frequent movement + encouraged to meet appropriate gestational weight gain recommendations by	Women assigned to the control group participated in informative group sessions imparted by study staff, receiving health advice about dental care and child safety.	BMI ≥ 25	31

				monitoring their diet, physical activity, and weight trajectory			
Uria-Minguito 2022	Spain	Europe	Physical Activity	Exercise program consisting of physical and emotional activation	Regular scheduled visits, usually every 4 – 5 weeks until the 36 – 38th week of gestation and then weekly until delivery.	All BMI groups	260
Vesco 2014	USA	North America	Mixed	Session included a nutrition and/or exercise topic, a behaviour change topic, and a goal-setting activity	Control participants in our study received a one-time advice session from the study dietician. They were given general information about healthy eating during pregnancy, without specific focus on the DASH dietary pattern or weight management. The dietician provided feedback on their food diaries and advised them to follow their obstetrical care providers' recommendations. It's important to note that our study did not provide routine prenatal care to either group of participants. The information provided through the study was given in addition to their regular medical care.	BMI ≥ 30	114
Wang 2016	China	Asia	Physical Activity	Supervised cycling program	Participants allocated to the control group continued with their usual daily activities.	BMI ≥ 25	265

Wang 2023	China	Asia	Diet	Dietary consultations offered monthly by dieticians	Usual Care	All BMI groups	519
Xu 2021	China	Asia	Physical Activity	Education on pregnancy weight management from obstetricians and had their diets assessed by nutritionists + physical activity according to the American College of Obstetricians and Gynecologists	Standard obstetrical care	All BMI groups	348
Xu 2023	China	Asia	Diet	Individual meal plans were given by nutritionists + balanced diet with vegetables, fruits, high-fibre whole grain products, low-fat dairy products, increased legumes, nuts and other plant proteins, and other plant proteins while avoiding foods rich in sugar and saturated fatty acids	Regular pregnancy check-ups	BMI ≥ 25	251

EVOO=Extra virgin olive oil, GWG = Gestational weight gain, HCP=Health Care Professionals, IOM = Institute of Medicine

Appendix 5: TIDieR intervention core components of individual participant data (IPD) studies in the meta-analysis of lifestyle interventions on gestational diabetes

Study name	Intervention type	Theory (Yes/No)	Resources	Structure	Method	Facilitators	Provider prior training	Location	Duration	Number of sessions	Ongoing support	Gestation week
Al Wattar 2019	Diet	Yes	None	Individual	Face-to-face	Allied health staff	Yes	Hospital/ Antenatal clinic	Moderate	Low	Yes	<20
Arthur 2020	Mixed	No	Self-monitoring tool	Individual	Face-to-face	NA	No/NR	Other	Moderate	Low	No	≥20
Assaf-Balut 2017	Diet	No	None	Group	Face-to-face	Allied health staff	No/NR	Hospital/ Antenatal clinic	Low	Low	No	<20
Baciuk 2008	Physical Activity	No	Other resource	Group	Face-to-face	Allied health staff	No/NR	Physical Activity Centre	High	High	No	<20
Barakat 2008	Physical Activity	No	Other resource	Group	Face-to-face	Allied health staff	Yes	Hospital/ Antenatal clinic	Moderate	High	Yes	≥20
Barakat 2011	Physical Activity	No	None	Group	Face-to-face	Allied health staff	No/NR	Hospital/ Antenatal clinic	High	High	No	<20
Barakat 2012a	Physical Activity	No	None	Group	Face-to-face	Allied health staff	No/NR	Hospital/ Antenatal clinic	High	High	No	<20
Barakat 2016	Physical Activity	No	None	Group	Face-to-face	Allied health staff	No/NR	Hospital/ Antenatal clinic	High	High	No	<20
Barakat 2018	Physical Activity	No	None	Group	Face-to-face	Allied health staff	No/NR	Hospital/ Antenatal clinic	High	High	No	<20

Bisson 2015	Physical Activity	No	None	Individual	Face-to-face	Allied health staff	No/NR	Physical Activity Centre	Low	High	No	<20
Bogaerts 2012	Mixed	Yes	Other resource	Group	Face-to-face	Medical staff	Yes	Hospital/ Antenatal clinic	Moderate	Low	No	<20
Bruno 2016	Mixed	No	Self-monitoring tool	Individual	Face-to-face	Allied health staff	No/NR	Hospital/ Antenatal clinic	High	Low	No	<20
Chao 2017	Mixed	Yes	Self-monitoring tool	Individual	Face-to-face + Remote	Allied health staff	Yes	Hospital/ Antenatal clinic	Moderate	High	Yes	<20
Cordero 2014	Physical Activity	No	None	Group	Face-to-face	Allied health staff	No/NR	Physical Activity Centre	High	High	No	<20
Dekker 2015	Physical Activity	No	Other resource	Individual	Face-to-face	Allied health staff	No/NR	Hospital/ Antenatal clinic	High	Low	Yes	<20
Dodd 2014	Mixed	Yes	Combination	Individual	Face-to-face	Allied health staff	Yes	Hospital/ Antenatal clinic	Moderate	Low	Yes	<20
Dodd 2019	Mixed	Yes	Combination	Individual	Face-to-face	Allied health staff	Yes	Hospital/ Antenatal clinic	Moderate	Low	Yes	<20
El Beltagy 2013	Mixed	No	Combination	NR	NR	NR	NR	NR	Low	NR	NR	<20
Garmendia 2020	Mixed	No	None	Individual	Face-to-face	Allied health staff	Yes	Hospital/ Antenatal clinic	High	High	No	<20
Garnaes 2016	Physical Activity	Yes	Self-monitoring tool	Group	Face-to-face	Allied health staff	No/NR	Hospital/ Antenatal clinic	Moderate	High	No	<20

Guelinckx 2010	Mixed	Yes	Other resource	Group	Face-to-face	Allied health staff	Yes	Hospital/ Antenatal clinic	Moderate	Low	No	<20
Harrison 2013	Mixed	Yes	Combination	Individual	Face-to-face	Allied health staff	No/NR	Hospital/ Antenatal clinic	Low	Low	No	<20
Hawkins 2014	Mixed	Yes	Combination	Individual	Face-to-face + Remote	Other	Yes	Hospital/ Antenatal clinic	High	Moderate	Yes	<20
Hui 2011	Mixed	No	Combination	Group	Face-to-face	Allied health staff	No/NR	Physical Activity Centre	Low	Moderate	No	≥20
Hui 2014	Mixed	No	Combination	Group	Face-to-face + Remote	Allied health staff	No/NR	Physical Activity Centre	Low	High	No	≥20
Jeffries 2009	Mixed	No	Self-monitoring tool	Individual	Face-to-face	Medical staff	No/NR	Hospital/ Antenatal clinic	High	Low	No	<20
Kennelly 2018	Mixed	Yes	Other resource	Group	Face-to-face	Allied health staff	No/NR	Hospital/ Antenatal clinic	High	Low	Yes	<20
Khaledan 2010	Physical Activity	No	None	Group	Face-to-face	Other	No/NR	Hospital/ Antenatal clinic	Low	High	No	≥20
Khoury 2005	Diet	No	Other resource	Individual	Face-to-face	Allied health staff	No/NR	Hospital/ Antenatal clinic	Moderate	Low	No	<20
Kunath 2019	Mixed	No	Combination	Individual	Face-to-face	Medical staff	Yes	Hospital/ Antenatal clinic	Moderate	Low	No	<20
Luoto 2011	Mixed	Yes	Other resource	Individual	Face-to-face	Medical staff	Yes	Hospital/ Antenatal clinic	High	Moderate	Yes	<20

McCarthy 2016	Diet	No	Combination	Individual	Face-to-face	Medical staff	No/NR	Hospital/ Antenatal clinic	Moderate	Low	Yes	≥20
Nascimento 2011	Physical Activity	No	Self-monitoring tool	Group	Face-to-face	Allied health staff	Yes	Hospital/ Antenatal clinic	Low	Moderate	No	≥20
Olson 2018	Mixed	Yes	Combination	Individual	Remote	NR	NA	Other	Moderate	NA	Yes	≥20
Ong 2009	Physical Activity	No	Other resource	Individual	Face-to-face	Other	No/NR	Other	Low	High	No	<20
Oostdam 2012	Physical Activity	No	None	Group	Face-to-face	Allied health staff	Yes	Hospital/ Antenatal clinic	Low	High	No	≥20
Peleaz 2019	Physical Activity	No	None	Group	Face-to-face	Allied health staff	No/NR	Hospital/ Antenatal clinic	High	High	No	<20
Perales 2014	Physical Activity	No	None	Group	Face-to-face	Allied health staff	No/NR	Hospital/ Antenatal clinic	High	High	No	<20
Petrella 2013	Mixed	No	Self-monitoring tool	Individual	Face-to-face	Allied health staff	No/NR	Hospital/ Antenatal clinic	High	Low	No	<20
Phelan 2011	Mixed	Yes	Combination	Individual	Face-to-face	Allied health staff	No/NR	Other	Moderate	Low	Yes	<20
Phelan 2018	Mixed	Yes	Combination	Individual	Face-to-face	Allied health staff	No/NR	Other	Moderate	Moderate	No	<20
Poston 2013	Mixed	Yes	Combination	Individual	Face-to-face + Remote	Allied health staff	Yes	Hospital/ Antenatal clinic	High	Moderate	Yes	≥20
Poston 2015	Mixed	Yes	Combination	Group	Face-to-face + Remote	Other	Yes	Hospital/ Antenatal clinic	Low	Moderate	Yes	<20

Rauh 2013	Mixed	Yes	Self-monitoring tool	Individual	Face-to-face	Other	Yes	Hospital/ Antenatal clinic	Low	Low	No	≥20
Renault 2013	Diet	No	Self-monitoring tool	Individual	Face-to-face	Allied health staff	No/NR	Hospital/ Antenatal clinic	High	Moderate	Yes	<20
Rono 2018	Mixed	No	None	Individual	Face-to-face	Medical staff	No/NR	Hospital/ Antenatal clinic	High	Low	No	<20
Ruiz 2013	Physical Activity	No	None	Group	Face-to-face	Allied health staff	No/NR	Physical Activity Centre	High	High	No	<20
Sagedal 2017	Mixed	No	Other resource	Group	Face-to-face + Remote	Allied health staff	Yes	Physical Activity Centre	Moderate	High	Yes	≥20
Stafne 2012	Physical Activity	No	None	Group	Face-to-face	Allied health staff	No/NR	Hospital/ Antenatal clinic	Low	Moderate	No	≥20
Vinter 2011	Mixed	Yes	Self-monitoring tool	Group	Face-to-face	Allied health staff	No/NR	Physical Activity Centre	Moderate	High	No	<20
Vitolo 2011	Diet	No	Other resource	Individual	Face-to-face	Other	No/NR	Hospital/ Antenatal clinic	Low	Low	No	≥20
Walsh 2012	Diet	No	Other resource	Group	Face-to-face	Allied health staff	No/NR	Hospital/ Antenatal clinic	Moderate	Low	No	<20
Willcox 2017	Mixed	Yes	Combination	Individual	Face-to-face + Remote	Allied health staff	No/NR	Hospital/ Antenatal clinic	Low	Moderate	Yes	≥20
Wolff 2008	Diet	No	None	Individual	Face-to-face	Allied health staff	Yes	Hospital/ Antenatal clinic	NAC	Moderate	No	<20

NR=Not reported

Appendix 6: Risk-of-bias assessment in IPD studies compared with non-IPD.

	Risk-of-bias rating, n (%)								
	Low			Unclear			High		
**Items**	**IPD (n=54)**	**Non-IPD (n=50)**	**All eligible studies (n=104)**	**IPD (n=54)**	**Non-IPD (n=50)**	**All eligible studies (n=104)**	**IPD (n=54)**	**Non-IPD (n=50)**	**All eligible studies (n=104)**
Random sequence generation	49 (91%)	38 (76%)	87 (84%)	5 (9%)	7 (14%)	12 (12%)	0	5 (11%)	5 (5%)
Allocation concealment	33 (61%)	28 (56%)	61 (59%)	18 (33%)	21 (42%)	39 (38%)	3 (6%)	1(2%)	4 (4%)
Blinding of participants and personnel	1 (2%)	0	1 (1%)	19 (35%)	25 (50%)	44 (42%)	34 (63%)	22 (50%)	59 (57%)
Blinding outcome assessment	22 (41%)	17 (34%)	39 (38%)	18 (33%)	30 (60%)	48 (46%)	14 (26%)	3 (6.5%)	17 (17%)
Incomplete outcome data	48 (89%)	43 (86%)	91 (88%)	0	2 (4%)	2 (2%)	6 (11%)	5 (11%)	11 (11%)
Selective outcome reporting	33 (61%)	32 (64%)	65 (63%)	13 (24%)	17 (34%)	30 (29%)	8 (15%)	1 (2%)	9 (9%)
Overall risk of bias	33 (61%)	34 (68%)	67 (64%)	0	0	0	21 (39%)	16 (33%)	37 (36%)

Appendix 7a: Effects of lifestyle interventions on gestational diabetes (any criteria) in studies at low or medium risk of bias

Intervention	Source	Number of studies	Number of women	OR (95% CI)	95% PI	Tau-squared
Physical activity	IPD	11	2,993	**0.59 (0.43, 0.82)**	**0.43, 0.82**	0.00 (0.00, 0.44)
	IPD+AD	22	6,967	**0.67 (0.55, 0.82)**	**0.55, 0.82**	0.00 (0.00, 0.20)
Diet	IPD	5	1,930	0.89 (0.69, 1.16)	0.66, 1.21	0.00 (0.00, 0.56)
	IPD+AD	10	2,818	0.93 (0.63, 1.38)	0.46, 1.87	0.06 (0.00, 0.86)
Mixed	IPD	17	10,624	**1.14 (1.01, 1.29)**	**1.01, 1.29**	0.00 (0.00, 0.09)
	IPD+AD	36	14,781	0.96 (0.85, 1.09)	0.65, 1.40	0.03 (0.00, 0.12)
All	IPD	33	15,547	0.94 (0.82, 1.08)	0.64, 1.39	0.03 (0.00, 0.13)
	IPD+AD	68	24,566	**0.87 (0.78, 0.97)**	0.55, 1.36	0.05 (0.01, 0.13)

IPD – Individual Participant Data; AD – Aggregate Data; OR – Odds Ratio; CI – Confidence Interval PI – Prediction Interval

Appendix 7b: Effects of lifestyle interventions on gestational diabetes (NICE definition) in IPD studies at low or medium risk of bias

Intervention	Source	Number of studies	Number of women	OR (95% CI)	95% PI	Tau-squared
Physical activity	IPD	3	123	0.25 (0.04, 1.58)	0, 55.47	0.00 (0.00, 23.81)
Diet	IPD	1	917	0.75 (0.53, 1.07)	-	0.00 (0.00, 0.00)
Mixed	IPD	9	5315	1.13 (0.97, 1.32)	0.97, 1.32	0.00 (0.00, 0.11)
All	IPD	13	6,355	1.01 (0.83, 1.22)	0.69, 1.48	0.02 (0.00, 0.18)

OR – Odds Ratio; CI – Confidence Interval PI – Prediction Interval

Appendix 8: Contour-enhanced funnel plots for the IPD meta-analysis of gestational diabetes defined by (a) any criteria and (b) NICE

(a)

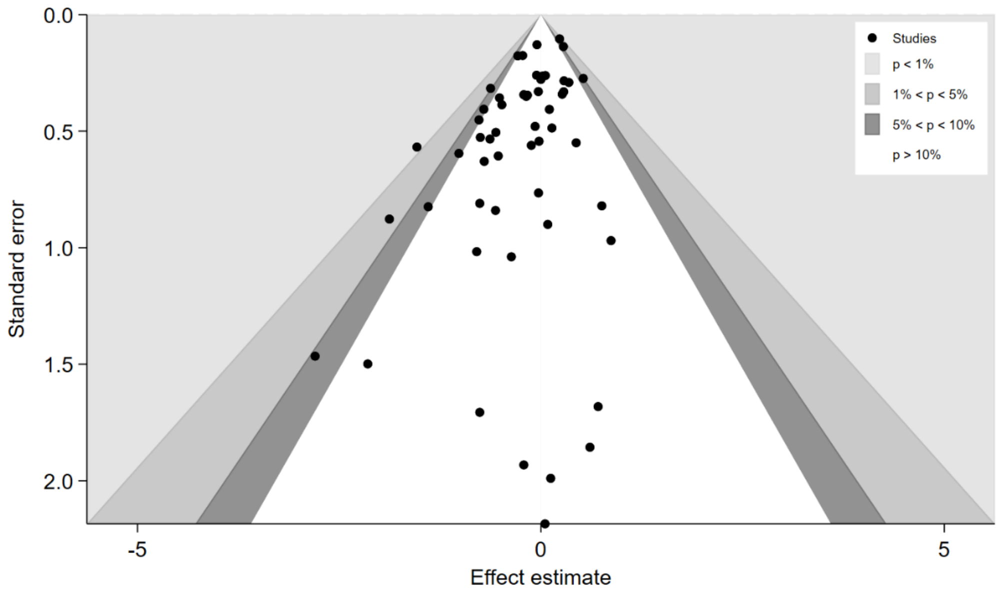

Contour-enhanced funnel plot for overall intervention effects on gestational diabetes as defined by any criteria (IPD only).

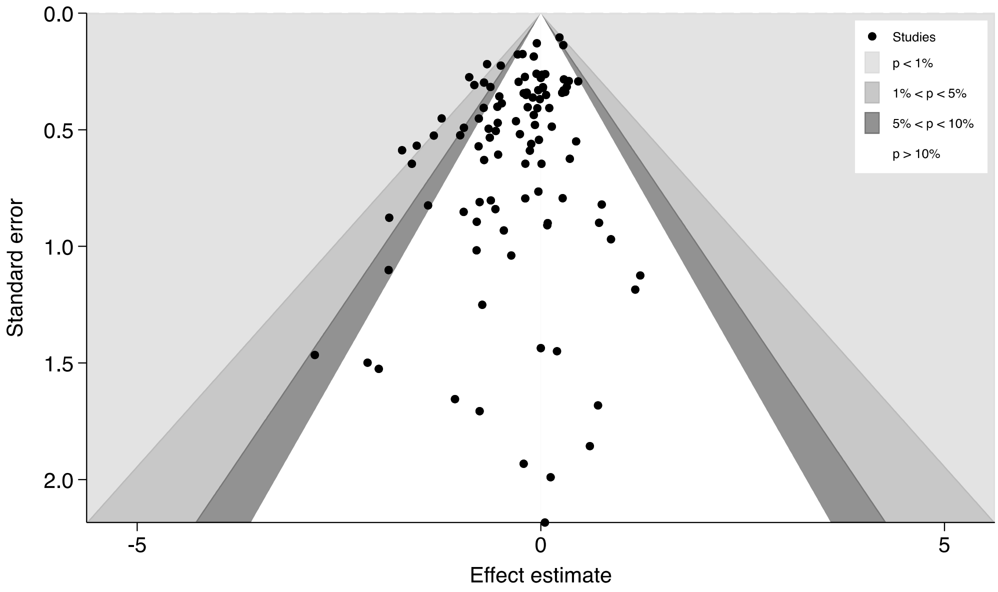

Contour-enhanced funnel plot for overall intervention effects on gestational diabetes as defined by any criteria (IPD and aggregate data).

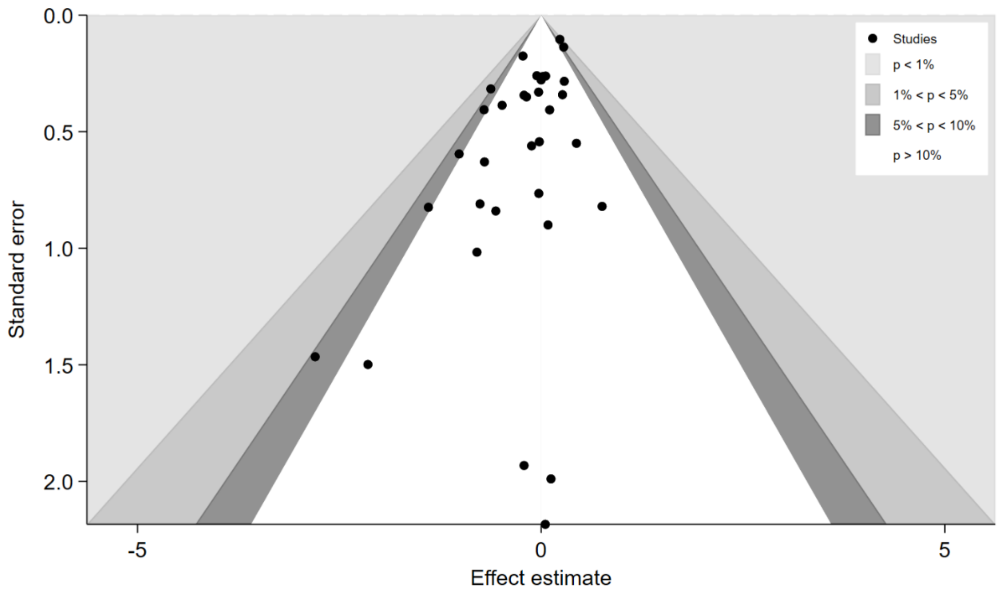

Contour-enhanced funnel plot for overall intervention effects on gestational diabetes as defined by any criteria (IPD studies classified as low risk of bias)

(b)

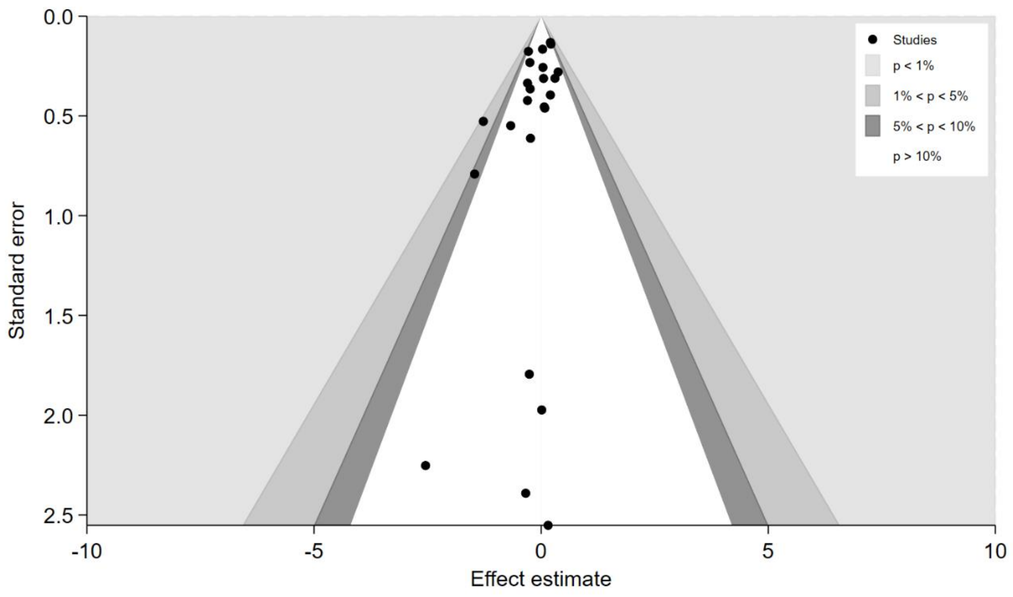

Contour-enhanced funnel plot for overall intervention effects on gestational diabetes as defined by NICE (IPD only).

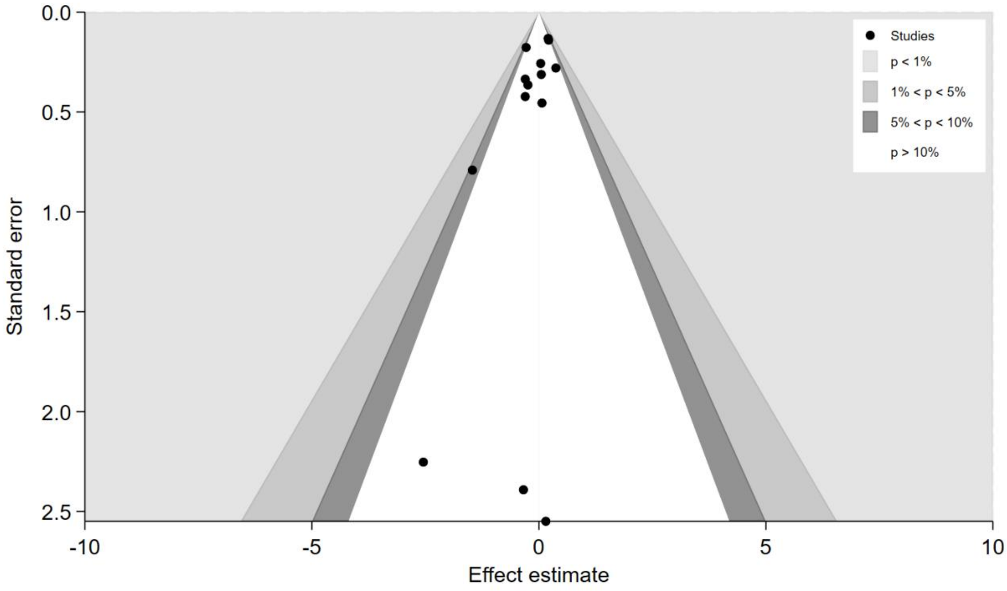

Contour-enhanced funnel plot for overall intervention effects on gestational diabetes as defined by NICE (IPD studies classified as low risk of bias).

Appendix 9: Association of overall lifestyle intervention TIDieR component subgroups with gestational diabetes.

Intervention core component	Number of IPD trials n (%)	OR	95% CI	Comparison p-value	Global p-value
**Structure**					
Individual	25 (48.1%)	**1.02**	**(0.89, 1.17)**	**Ref**	-
Group	27 (51.9%)	**0.81**	**(0.68, 0.97)**	**0.048**	
**Number of sessions**					
Low + moderate	20 (39.2%)	0.95	(0.82, 1.11)	(ref)	-
High	31 (60.8%)	0.80	(0.62, 1.03)	0.230	
**Theory**					
No	35 (66.0%)	0.86	(0.73, 1.01)	Ref	-
Yes	18 (34.0%)	1.00	(0.83, 1.20)	0.230	
**Resources**					
Combination + Other resources	17 (32.1%)	1.03	(0.88, 1.21)	Ref	0.100
None	26 (49.0%)	0.85	(0.69, 1.04)	0.135	
Self-Monitoring tool	10 (18.9%)	0.72	(0.52, 1.00)	0.057	
**Method**					
Face to face	45 (88.2%)	0.90	(0.79, 1.03)	Ref	-
Face to face + Remote	6 (11.8%)	1.00	(0.71, 1.41)	0.593	
**Facilitator**					
Allied health staff	39 (76.0%)	0.84	(0.72, 0.98)	Ref	0.204
Medical staff	6 (12.0%)	1.14	(0.85, 1.53)	0.076	
Other	6 (12.0%)	0.91	(0.59, 1.38)	0.745	
**Provider prior training**					
No/NR	33 (64.7%)	0.82	**(0.69, 0.96)**	**Ref**	-
Yes	18 (35.3%)	1.04	**(0.90, 1.20)**	**0.031**	
**Location**					
Hospital/ Antenatal clinic	39 (75.0%)	0.92	(0.81, 1.05)	Ref	0.445
Exercise centre	8 (15.4%)	0.77	(0.51, 1.15)	0.385	
Other	5 (9.6%)	1.10	(0.74, 1.66)	0.401	
**Duration**					
Moderate	18 (34.6%)	1.03	(0.86, 1.24)	Ref	0.228
High	20 (38.5%)	0.85	(0.69, 1.05)	0.174	
Low	15 (26.9%)	0.81	(0.62, 1.05)	0.134	
**Ongoing support**					
No	36 (69.2%)	0.86	(0.73, 1.00)	Ref	-
Yes	16 (30.8%)	1.01	(0.84, 1.21)	0.172	
**Gestational age at randomisation**					
<20	39 (73.6%)	0.90	(0.79, 1.04)	Ref	-
≥20	14 (26.4%)	0.96	(0.75, 1.23)	0.674	

IPD – Individual Participant Data; OR – Odds Ratio; CI – Confidence Interval

Appendix 10: Estimated probabilities (%) of each intervention having each rank & the mean rank with 95% confidence intervals for each intervention.

Rank (%)	Intervention<br>Physical activity	Diet	Control	Mixed
Best	89	11	0	0
2nd	11	83.1	3.4	2.5
3rd	0	3.1	78	18.9
Worst	0	2.8	18.6	78.6
Mean Rank (95% CI)	1.1 (1 to 2)	2 (1 to 3)	3.2 (2 to 4)	3.8 (3 to 4)
SUCRA (%)	96.3	67.4	28.3	8
